# Immunogenicity and Protective Efficacy of Radiation-Attenuated and Chemo-Attenuated PfSPZ Vaccines in Equatoguinean Adults

**DOI:** 10.4269/ajtmh.20-0435

**Published:** 2020-11-16

**Authors:** Said A. Jongo, Vicente Urbano, L. W. Preston Church, Ally Olotu, Stephen R. Manock, Tobias Schindler, Ali Mtoro, Natasha KC, Ali Hamad, Elizabeth Nyakarungu, Maximillian Mpina, Anna Deal, José Raso Bijeri, Martin Eka Ondo Mangue, Beltrán Ekua Ntutumu Pasialo, Genaro Nsue Nguema, Salomon Nguema Owono, Matilde Riloha Rivas, Mwajuma Chemba, Kamaka R. Kassim, Eric R. James, Thomas C. Stabler, Yonas Abebe, Elizabeth Saverino, Julian Sax, Salome Hosch, Anneth-Mwasi Tumbo, Linda Gondwe, J. Luis Segura, Carlos Cortes Falla, Wonder Philip Phiri, Dianna E. B. Hergott, Guillermo A. García, Christopher Schwabe, Carl D. Maas, Tooba Murshedkar, Peter F. Billingsley, Marcel Tanner, Mitoha Ondo’o Ayekaba, B. Kim Lee Sim, Claudia Daubenberger, Thomas L. Richie, Salim Abdulla, Stephen L. Hoffman

**Affiliations:** 1 Ifakara Health Institute, Bagamoyo Research and Training Centre, Bagamoyo, Tanzania;; 2 Ministry of Health and Social Welfare, Government of Equatorial Guinea, Bioko Norte, Equatorial Guinea;; 3 Sanaria Inc., Rockville, Maryland;; 4 Swiss Tropical and Public Health Institute, Basel, Switzerland;; 5 Protein Potential LLC, Rockville, Maryland;; 6 Medical Care Development International, Silver Spring, Maryland;; 7 Marathon EG Production, Ltd., Bioko Norte, Equatorial Guinea

## Abstract

*Plasmodium falciparum* sporozoite (PfSPZ) Vaccine (radiation-attenuated, aseptic, purified, cryopreserved PfSPZ) and PfSPZ-CVac (infectious, aseptic, purified, cryopreserved PfSPZ administered to subjects taking weekly chloroquine chemoprophylaxis) have shown vaccine efficacies (VEs) of 100% against homologous controlled human malaria infection (CHMI) in nonimmune adults. *Plasmodium falciparum* sporozoite-CVac has never been assessed against CHMI in African vaccinees. We assessed the safety, immunogenicity, and VE against homologous CHMI of three doses of 2.7 × 10^6^ PfSPZ of PfSPZ Vaccine at 8-week intervals and three doses of 1.0 × 10^5^ PfSPZ of PfSPZ-CVac at 4-week intervals with each arm randomized, double-blind, placebo-controlled, and conducted in parallel. There were no differences in solicited adverse events between vaccinees and normal saline controls, or between PfSPZ Vaccine and PfSPZ-CVac recipients during the 6 days after administration of investigational product. However, from days 7–13, PfSPZ-CVac recipients had significantly more AEs, probably because of Pf parasitemia. Antibody responses were 2.9 times higher in PfSPZ Vaccine recipients than PfSPZ-CVac recipients at time of CHMI. Vaccine efficacy at a median of 14 weeks after last PfSPZ-CVac dose was 55% (8 of 13, *P* = 0.051) and at a median of 15 weeks after last PfSPZ Vaccine dose was 27% (5 of 15, *P* = 0.32). The higher VE in PfSPZ-CVac recipients of 55% with a 27-fold lower dose was likely a result of later stage parasite maturation in the liver, leading to induction of cellular immunity against a greater quantity and broader array of antigens.

## INTRODUCTION

Despite an international investment in malaria control of more than $4 billion annually, the numbers of deaths and clinical cases of malaria were essentially unchanged from 2015 to 2018.^1,2^ Depending on the estimate,^1,3^ there are 16,730–28,000 deaths from malaria every 2 weeks. The Bioko Island Malaria Elimination Program has been working to reduce the impact of malaria on Bioko Island, Equatorial Guinea, for 15 years. During that period, the prevalence of malaria in 2- to 14-year-olds and the deaths attributed to malaria have been reduced by 73% and 85%, respectively.^4^ However, despite an annual investment of ∼$30 per capita in malaria control efforts by this team of Equatoguineans and international experts, the prevalence of malaria in 2- to 14-year-olds has been unchanged for the past 6 years, paralleling the international situation (G. A., Garcia, personal communication). New tools are required.^5^ We believe introduction of an effective malaria vaccine would be the most efficient way to decrease and eventually halt malaria transmission and eliminate the disease from Bioko Island.^6^

We have been assessing Sanaria’s whole *Plasmodium falciparum* sporozoite (PfSPZ) vaccines for more than 9 years.^7–19^ There are no vaccines with marketing authorization (licensure) against diseases caused by parasites in humans, and there have previously been no vaccines against human infectious diseases composed of eukaryotic cells. With little to no human experience to draw on, the optimization of vaccination regimens with PfSPZ vaccines has been empirical. Here, we report the safety, immunogenicity, and vaccine efficacies (VE) against controlled human malaria infection (CHMI) of Sanaria^®^ PfSPZ Vaccine (radiation-attenuated PfSPZ)^7,8,10–12,14–19^ and PfSPZ-CVac (infectious PfSPZ Challenge administered to subjects taking chloroquine chemoprophylaxis)^9,13^ in healthy 18- to 35-year-old Equatoguinean adults.

## MATERIALS AND METHODS

### Study design and population.

This age de-escalation, double-blind, randomized, placebo-controlled trial was conducted in Baney, Equatorial Guinea, between October 2016 and January 2018. It had two major components: an age de-escalation and age escalation component to assess safety and immunogenicity of PfSPZ Vaccine in 6 months to 17-year-olds and 36- to 65-year-olds (part A) and a safety, immunogenicity, and CHMI component to assess VE in 18- to 35-year-olds of PfSPZ Vaccine and PfSPZ-CVac (part B); part B is described in this report.

For part B, healthy male and female subjects aged 18–35 years were recruited from the Baney district and city of Malabo on Bioko Island. Fifty subjects who met inclusion and exclusion criteria (Supplemental Appendix, Tables S1 and S2) and successfully completed a test of understanding were consented and enrolled. The eligibility criteria are available at https://clinicaltrials.gov/show/NCT02859350. Subjects were allocated to either the PfSPZ Vaccine arm or the PfSPZ-CVac arm; within each arm, they were randomized to either vaccine or normal saline (NS). Controls (placebo subjects) in the PfSPZ-CVac arm also received chloroquine on the same schedule as did vaccinees.

### Investigational products (IP).

Sanaria PfSPZ Vaccine comprised radiation attenuated, aseptic, purified, vialed, cryopreserved PfSPZ.^7,8,10–12,14–20^ Sanaria PfSPZ Challenge is identical to PfSPZ Vaccine, except it is not attenuated.^9,13,21–29^ Normal saline was the placebo. Chloroquine phosphate (Resochín, Kern Pharma, Barcelona, Spain), administered weekly beginning 2 days before the first dose through to 12 days after the final dose, was used to chemo-attenuate PfSPZ Challenge for PfSPZ-CVac.

### Randomization and intervention.

**Figure 1.**
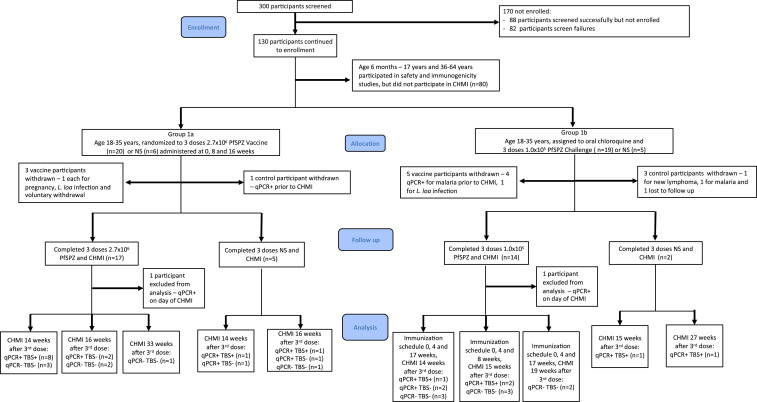
CONSORT diagram: Adult subjects aged 18–35 years. This figure appears in color at www.ajtmh.org.

Group 1a subjects were randomized to receive PfSPZ Vaccine (2.7 × 10^6^ PfSPZ) (*n* = 20) or NS (*n* = 6) at 0, 8, and 16 weeks. This dose, which was also being assessed at the same time in Burkina Faso (NCT02663700), was chosen assuming higher doses would be associated with increased immunogenicity and protection. Group 1b, PfSPZ-CVac, subjects were randomized to receive PfSPZ Challenge (1.0 × 10^5^ PfSPZ) (*n* = 19) or NS (*n* = 5) at 0, 4, and 8 weeks; PfSPZ Challenge and corresponding NS recipients received chloroquine. The dosing intervals for both groups were the same as in previous trials of PfSPZ Vaccine^12,16–18,30^ and PfSPZ-CVac.^9,13^ The study team was blinded to treatment assignment within each group. *Plasmodium falciparum* sporozoite Vaccine, PfSPZ Challenge, or NS in 0.5 mL was administered by DVI through a 25-gauge needle. Chloroquine was administered orally under direct observation 2 days before the first dose of PfSPZChallenge or NS in the PfSPZ-CVac group and weekly thereafter through 5 days after the final injection of PfSPZChallenge or NS (Figure 1, Supplemental Figure S1); the first dose was 600 mg chloroquine base, and subsequent doses were 300 mg chloroquine base.

### Vaccine efficacy.

Vaccine efficacy was assessed by CHMI by DVI of 3.2 × 10^3^ PfSPZ of PfSPZ Challenge and calculated based on the first positive quantitative PCR result. Controlled human malaria infection were planned for 10–14 weeks after last immunization, although for several subjects, the CHMI was delayed (Figure 1 and Supplemental Figure S1). Subjects were observed as inpatients beginning 8 days after PfSPZ Challenge injection until diagnosed by thick blood smear (TBS) and treated, or until day 21. Thick blood smear-negative subjects continued with every other day outpatient monitoring until day 28. After initiation of treatment, TBSs were assessed until two consecutive daily TBSs were negative. A qPCR specimen was obtained at each study visit during CHMI and at the final scheduled study visit (56 days after CHMI). All qPCR samples were run retrospectively, unless to confirm a positive TBS, in which case they were run within 24 hours.

### Adverse events (AE).

Solicited local (Supplemental Table S3) AEs were collected for 3 days after each immunization. Solicited systemic (Supplemental Table S3) and unsolicited AEs were collected for 7 and 28 days, respectively, after each immunization in Group 1a. In Group 1b to account for AEs that might be related to chloroquine administration or the transient parasitemia associated with PfSPZ-CVac, solicited AEs were collected from the first day of chloroquine administration (2 days before the first immunization) through 12 days after final immunization and 7 days after final chloroquine dose (Supplemental Table S4). Solicited AEs after injection of PfSPZ Challenge for CHMI were recorded for 5 days. Subjects were observed for 2 hours after administration of PfSPZ Vaccine or PfSPZ Challenge, then followed with daily home or clinic visits. Any subject who reported AEs at home was referred to the clinic. Grading of severity of AEs and relatedness to IP were carried out according to a prespecified system (Supplemental Table S3). Subjects were admitted to a hotel 8 days after PfSPZ Challenge administration for CHMI to be observed and treated for Pf malaria as needed. Symptoms and signs identified through prespecified (Supplemental Table S3) and open-ended questioning during the 8- to 28-day period were assessed for relationship to Pf infection and considered related if the event was within 3 days before and 7 days after the TBS was first positive.

### Treatment.

*Plasmodium* sp. infections diagnosed in subjects before CHMI were treated according to national guidelines with artesunate–amodiaquine or artemether–lumefantrine. Subjects with positive TBSs in the 28-day interval following CHMI were treated with AL within 24 hours of first positive TBS confirmed by qPCR. Subjects who were TBS negative were treated at the final study visit on day 56 after CHMI, regardless of qPCR findings.

### Detection of Pf parasites and parasite DNA.

After each immunizing dose in the PfSPZ-CVac arm of the study, parasitemia was monitored daily on days 6–10 by TBS and qPCR. During CHMI, samples were assessed by TBS and qPCR twice daily on days 8–14 after injection of PfSPZ Challenge, daily thereafter until positive or until day 20 and on days 22, 24, 26, and 28. Thick blood smears could be performed more frequently, if subjects had symptoms or signs consistent with malaria.

Slide preparation and reading for TBSs were performed as described.^24^ In brief, 10 µL of blood collected in EDTA was placed on a 10-mm by 20-mm rectangle on a glass slide, dried, and stained. For asymptomatic individuals, ∼0.5 µL of blood was assessed. For symptomatic individuals, ∼2.0 µL of blood was assessed. Two asexual erythrocytic stage Pf parasites had to be identified for a slide to be considered positive, yielding a lower limit of detection for a positive slide of four parasites/µL blood when ∼0.5 µL of blood was assessed.

Parasites were quantified by qPCR using the PlasQ qPCR assay as described.^31^ The lower limit of detection for this qPCR assay was 50 copies/mL.

A single positive time point was considered positive for infection with Pf. After the start of CHMI, the time of the first blood sample positivity by qPCR was used to determine infection status and calculation of the prepatent period. During CHMI, all samples were analyzed by qPCR in real time as they were continuously collected from the subjects. Samples collected during the immunization period were analyzed retrospectively.

We used molecular approaches to discriminate between NF54, the vaccine, and CHMI strain and naturally acquired infections. First, *Plasmodium* species differentiation by qPCR was conducted.^31^ Samples positive for Pf were genotyped by assessing polymorphisms for merozoite surface protein 1/merozoite surface protein 2^32^ and selected microsatellite markers.^33^ In addition, two widespread markers of sulphadoxine–pyrimethamine, dihydrofolate reductase and deoxyhypusine synthase, were amplified and sequenced.^34^

### Chloroquine levels.

Whole blood stored at −80°C was shipped to the Clinical Pathology Department, Noguchi Memorial Institute for Medical Research, University of Ghana. Samples collected on the day of first administration of IP, which was 2 days after administration of the loading dose of chloroquine (600 mg base), were analyzed in a blinded fashion for chloroquine, using high-performance liquid chromatography. The samples were run in two lots, corresponding to the two different cohorts enrolled in the PfSPZ-CVac arm of the trial. For the second cohort, plasma samples from the same time point stored at −80°C were also analyzed by Swiss BioQuant AG, Reinach, Switzerland, using HPLC.

### Antibody assays.

Blood for immunogenicity testing was drawn before the first immunization, 2 weeks after final immunization, and before CHMI. Serum was separated and frozen at −80°C within 4 hours of collection. IgG antibodies to Pf circumsporozoite protein (PfCSP) and Pf merozoite surface protein 1 (PfMSP1) were assessed by ELISA as described.^17^ The serum dilution at which optical density was 1.0 (OD 1.0), the difference between the post-OD 1.0 and pre-OD 1.0 (net OD 1.0), and the ratio of post-OD 1.0 to pre-OD 1.0 (OD 1.0 ratio) were calculated. An individual was considered to have seroconverted if the net OD 1.0 was ≥ 50 and the OD 1.0 ratio was ≥ 3.0.

### Statistical analysis.

The sample size of 20 vaccinees per dosage group with six controls was chosen to show with a power of 80% that a 40% Pf infection frequency in vaccinees was significantly different from a 99% Pf infection frequency in controls (α = 0.05, two tailed), with allowance for loss of up to two vaccinees and one control. Categorical variables were summarized using absolute (*n*) and relative (%) frequencies. Continuous variables were summarized using mean and SD, median, and range. Comparisons of categorical variables between groups were analyzed using Barnard’s two-sided exact unconditional test; for comparisons of continuous variables, the Mann–Whitney two-sided test was used. Time to event was assessed by Kaplan–Meier curves and logrank for trend. Time to event data was analyzed from CHMI injection until positive qPCR result. No corrections were made for multiple comparisons because of the early phase nature of this trial. A *P* value < 0.05 was considered significant.

## RESULTS

### Vaccine efficacy.

#### Normal saline controls.

Four of 11 subjects did not undergo CHMI (Figure 1). Two subjects developed malaria before CHMI, one was withdrawn with a new diagnosis of non-Hodgkin lymphoma and one was lost to follow-up. Four of the remaining seven saline controls were positive by both TBS and qPCR after CHMI, two were negative by TBS but positive by qPCR, and one was negative by both tests after CHMI (Table 1). This last individual did not receive chloroquine or any other antimalarial during the study.

**Table 1:** Vaccine efficacy against homologous CHMI

	# Undergoing CHMI	Median time from last vaccine dose to CHMI (range)	# Without parasitemia at 28 days by TBS	# Without parasitemia at 28 days by qPCR	VE by qPCR
PfSPZ vaccine	17*	14 weeks (14–33 weeks)	8	6	27% (*P* = 0.32)†
PfSPZ-CVac	14*	15 weeks (14–19 weeks)	10	8	55% (*P* = 0.051)†
Controls (pooled)	7	–	3	1	–

CHMI = controlled human malaria infection; VE = vaccine efficacies.

* One subject was excluded from analysis in the PfSPZ Vaccine arm and one was excluded in the PfSPZ-CVac arm after they were found to have naturally acquired Pf parasitemia by qPCR on the day of CHMI.

† *P*-values calculated using Barnard’s test, two tailed.

#### PfSPZ vaccine.

Three of 20 subjects did not undergo CHMI. One subject became pregnant during immunization, one did not respond to initial treatment for incidental *Loa loa* infection, and one withdrew. Seventeen subjects immunized with 2.7 × 10^6^ PfSPZ of PfSPZ Vaccine underwent CHMI. One subject was excluded from analysis after he was retrospectively determined to be qPCR-positive for Pf on the day of CHMI. Fifteen PfSPZ Vaccine subjects underwent CHMI 14–16 (median 14) weeks after last vaccine dose. Eight were positive by both TBS and qPCR, two were negative by TBS but positive by qPCR, and five were negative by both TBS and qPCR. The 16th subject immunized with PfSPZ Vaccine underwent first CHMI 33 weeks after last immunization and was negative by both TBS and qPCR (Table 1). Vaccine efficacies at a median of 14 weeks after last dose of vaccine was 27% (6 of 16 vaccinees versus one of seven controls negative by qPCR, *P* = 0.32, Barnard’s test, two tailed).

#### PfSPZ-CVac.

**Figure 2.**
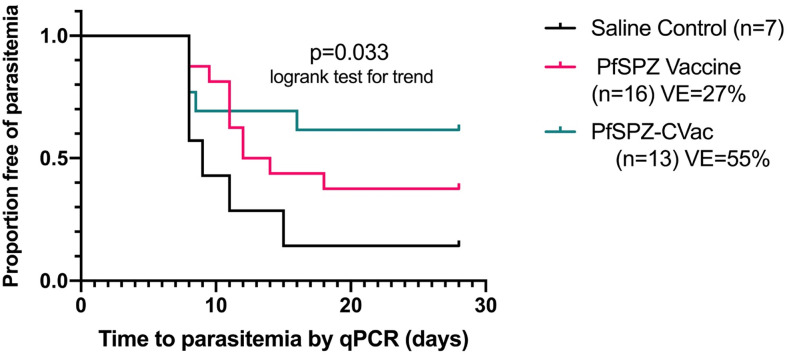
Kaplan–Meier survival curves in vaccinees and controls as assessed by qPCR. Kaplan–Meier curves in subjects undergoing controlled human malaria infection (CHMI) after the last of three doses with 2.7 × 10^6^ PfSPZ (*n* = 16) or 1.0 × 10^5^ PfSPZ Challenge (*n* = 13) vs. pooled saline (*n* = 5) and oral chloroquine plus saline controls (*n* = 2). The median time from the last dose to CHMI was 14 weeks for PfSPZ Vaccine and 15 weeks for PfSPZ-CVac. This figure appears in color at www.ajtmh.org.

Five of 19 subjects did not undergo CHMI. One subject developed *L. loa* infection during immunization, and four were positive for Pf by qPCR before CHMI. Fourteen subjects immunized with PfSPZ-CVac underwent CHMI. One subject was excluded from analysis because he was retrospectively determined to be qPCR-positive for Pf on the day of CHMI. Thirteen PfSPZ-CVac subjects underwent CHMI 14–19 (median, 15) weeks after last vaccine dose. Three were positive by both TBS and qPCR, two were negative by TBS but positive by qPCR, and eight were negative by both TBS and PCR (Table 1). Vaccine efficacies at a median of 15 weeks after last vaccine dose was 55% (8 of 13 vaccinees negative versus one of seven controls negative by qPCR, *P* = 0.051). By time-to-event analysis (Figure 2), there was a significant trend toward improved VE (*P* = 0.033, logrank test for trend) from saline controls to PfSPZ Vaccine to PfSPZ-CVac. The VEs of PfSPZ-CVac (55%) and PfSPZ Vaccine (27%) were not significantly different (*P* = 0.27).

#### Prepatent periods.

The prepatent periods for qPCR-positive and TBS-positive subjects are presented in Table 2. There were no significant differences in the prepatent periods by qPCR, although the results for qPCR may be skewed because several subjects were positive on the first qPCR measurement taken on day 8 after CHMI and may have been positive earlier. By TBS, the prepatent periods were significantly longer for PfSPZ Vaccine than controls (*P* = 0.02), but not for PfSPZ-CVac recipients who became parasitemic.

**Table 2:** Prepatent periods by qPCR and TBS

	PfSPZ vaccine	PfSPZ-CVac	Controls
Controlled human malaria infection (*n*), evaluable	16	13	7
qPCR + (*n*)	10	5	6
Prepatent period, qPCR (days)			
Median	11.0	8.0	8.5
Minimum, maximum	8, 18	8, 16	8, 15
*P*-value (vs. control)	*P* = 0.21	*P* = 0.84	–
TBS+ (*n*)	8	3	4
Prepatent period, TBS (days)			
Median	17	14	14.5
Minimum, maximum	15, 19	14, 26	13, 16
*P*-value (vs. control)	*P* = 0.02	*P* = 0.89	–

PfSPZ = *Plasmodium falciparum* sporozoite; TBS = thick blood smear.

### Antibody responses.

**Figure 3.**
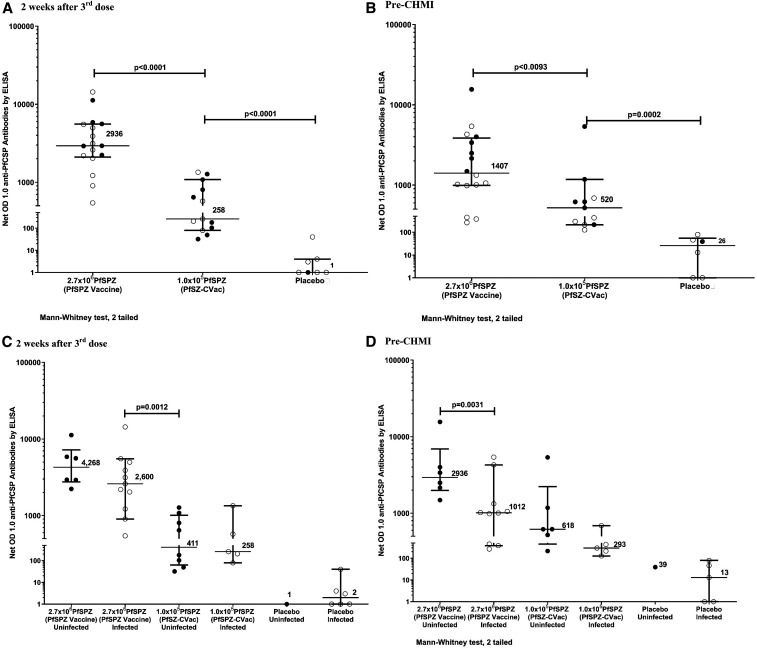
Antibodies to *Plasmodium falciparum* circumsporozoite protein (PfCSP) ELISA. IgG antibodies to Pf circumsporozoite protein PfCSP by ELISA 2 weeks after the third dose (**A**) and at the time of controlled human malaria infection (CHMI) (**B**) comparing PfSPZ Vaccine and PfSPZ-CVac. IgG antibodies to Pf circumsporozoite protein PfCSP by ELISA 2 weeks after the third dose (**C**) and at the time of CHMI (**D**) comparing infected and uninfected subjects in PfSPZ Vaccine and PfSPZ-CVac. Filled circles (•) represent subjects remaining uninfected after CHMI; open circles (◯) represent subjects infected after CHMI. Additional figures for antibodies to PfCSP and the antibody results for MSP-1 are found in the Supplemental Appendix.In the PfSPZ Vaccine group, 18/18 (100%), and in PfSPZ-CVac group, 7/17 (41.2%) subjects seroconverted (*P* = 0.00012) when measured 2 weeks after the third dose. When PfCSP antibodies were measured before CHMI, 15/16 (93.8%) had positive antibody response in the PfSPZ Vaccine group and 8/13 (61.5%) in the PfSPZ-CVac group (*P* = 0.038) (Supplemental Table 5). Antibody responses to PfCSP 2 weeks after the third dose (**A**) were significantly higher in the PfSPZ Vaccine group (median net OD 1.0 = 2,936) than in the PfSPZ-CVac group (median net OD 1.0 = 258) (*P* < 0.0001, Wilcoxon signed-rank test, two tailed). The PfSPZ-CVac group had higher antibody levels than normal saline (NS) controls 2 weeks after the third dose (median net OD 1.0 = 1) (net *P* < 0.0001, Wilcoxon signed-rank test, two tailed). Antibody responses to PfCSP the day before CHMI (**B**) were significantly higher in the PfSPZ Vaccine group (median net OD 1.0 = 1,407) than in the PfSPZ-CVac group (median net OD 1.0 = 520) (net *P* = 0.0093, Mann–Whitney test, two tailed). The PfSPZ-CVac group had higher antibody levels than NS controls before CHMI (**B**, median net OD 1.0 = 26, *P* = 0.0002, Wilcoxon signed-rank test, two tailed). Median net OD 1.0 of PfCSP antibodies measured 2 weeks after the third dose (**C**) in the PfSPZ Vaccine group were higher in uninfected vs. that in infected subjects (median net OD 1.0 4,268 vs. 2,600, *P* = 0.180, Wilcoxon signed-rank test, two tailed), but the difference was not significant. Likewise, there was no significant difference in antibody levels 2 weeks after the third dose between subjects who received PfSPZ-CVac who were not infected, vs. those who became infected (median net OD 411 vs. 258, *P* = 0.833). There was a significant difference in net OD 1.0 anti-PfCSP antibody levels before CHMI (**D**) between subjects who received PfSPZ Vaccine who were uninfected vs. those who were infected (median net OD 1.0 2,936 vs. 1,012, *P* = 0.031, Wilcoxon signed-rank test, two tailed). Net OD 1.0 anti-PfCSP antibody levels before CHMI in subjects who received PfSPZ-CVac who were uninfected were higher than infected subjects (median net OD 618 vs. 293, *P* = 0.126), but not significantly.

Antibodies against PfCSP were assessed in subjects from all groups 14 days after the third immunization and the day before CHMI, which was 98–231 days after last immunization. We also assessed pre-CHMI sera for antibodies to the late liver stage/asexual erythrocytic stage protein PfMSP1 by ELISA (Figure 3, Supplemental Figure S2, Table S5).

#### Antibodies to PfCSP.

##### PfSPZ vaccine versus PfSPZ-CVac versus NS placebo.

Antibody responses to PfCSP 2 weeks after the third dose (Figure 3A, Supplemental Figure S2A, Table S5) were significantly higher in the PfSPZ Vaccine group (median net OD 1.0 = 2,936 and median OD 1.0 ratio = 40.35) than that in the PfSPZ-CVac group (median net OD 1.0 = 258 and median OD 1.0 ratio = 2.98) (net *P* < 0.0001 and ratio *P* < 0.0001, Mann–Whitney test, two tailed). The PfSPZ-CVac group had higher antibody levels than NS controls (median net OD 1.0 = 1 and median OD 1.0 ratio = 1.02) (net *P* < 0.0001 and ratio *P* = 0.0003). Antibody responses to PfCSP the day before CHMI (Figure 3B and Supplemental Figure S2B) were significantly higher in the PfSPZ Vaccine group (median net OD 1.0 = 1,407 and median OD 1.0 ratio 45.37) than in the PfSPZ-CVac group (median net OD 1.0 = 520 and median OD 1.0 ratio 4.07) (net *P* = 0.0093 and ratio *P* < 0.0001). The PfSPZ-CVac group had higher antibody levels than NS controls (median net OD 1.0 = 26 and median OD 1.0 ratio = 1.27) (net *P* = 0.0002 and ratio *P* < 0.0001).

##### Uninfected versus infected 2 weeks after third dose.

There was no significant difference in antibody levels 14 days after the third dose between subjects who received PfSPZ Vaccine and did not become infected, versus those who became infected (median net OD 1.0 4,268 versus 2,600, *P* = 0.180 and median OD 1.0 ratio 67.57 versus 40.35, *P* = 0.591) (Figure 3C, Supplemental Figure S2C). Likewise, there was no significant difference in antibody levels 14 days after the third dose between subjects who received PfSPZ-CVac who were not infected, versus those who became infected (median net OD 411 versus 258, *P* = 0.833 and median OD 1.0 ratio 3.76 versus 2.48, *P* = 0.943). Antibody levels were higher in subjects who received PfSPZ Vaccine and became infected, versus those who received PfSPZ-CVac and did not become infected (median net OD 2600 versus 411, *P* = 0.0012 and median OD 1.0 ratio 40.35 versus 3.76, *P* = 0.0008).

##### Uninfected versus infected before CHMI.

There was a significant difference in net OD 1.0 antibody levels before CHMI between subjects who received PfSPZ Vaccine who were uninfected versus those who were infected (median net OD 1.0 2,936 versus 1,012, *P* = 0.031) (Figure 3D and Supplemental Figure S2D). The median OD 1.0 ratio was also higher in uninfected vaccinees but did not reach the level of statistical significance (median OD 1.0 ratio 60.28 versus 30.97, *P* = 0.219). In subjects who received PfSPZ-CVac who were uninfected or infected, the difference in the net OD 1.0 and OD 1.0 ratios was higher in the uninfected, but not significant (median net OD 618 versus 293, *P* = 0.126, and median OD 1.0 ratio 6.04 versus 2.49, *P* = 0.247).

#### Antibodies to PfMSP1.

##### *Uninfected* versus *infected before CHMI*.

In subjects who received PfSPZ Vaccine who were uninfected, the PfMSP1 median OD 1.0 measured before CHMI was higher than that of infected subjects (median OD 1.0 = 899 versus 55), but not significantly (*P* = 0.515) (Supplemental Figure S2E and Table S5). Uninfected subjects who received PfSPZ-CVac also had higher antibodies to PfMSP-1 before CHMI than the infected subjects (median OD 1.0 = 3,000 versus 423), but the difference was not significant (*P* = 0.178).

### Safety.

#### Solicited AEs following immunization.

##### PfSPZ vaccine.

**Figure 4.**
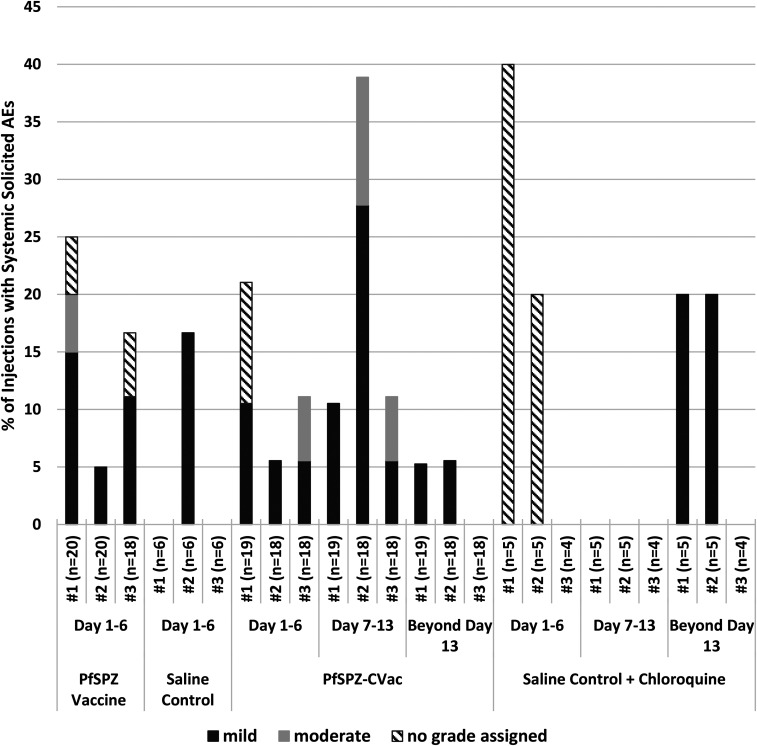
Solicited systemic adverse events as a percentage of doses administered. For subjects in the PfSPZ Vaccine arm, solicited adverse events (AEs) are reported for 6 days after each immunization. In the PfSPZ-CVac arm, solicited AEs are collected at the time of the first chloroquine administration (2 days before the first immunization) and continued for 12 days after the third immunization (70 days total). These AE are further categorized into predetermined intervals (days 1–6, 7–13, and beyond day 13) following each immunization (#1, #2, or #3) for PfSPZ-CVac. During days 1–6, reactions to vaccination were assessed, while during days 7–13 the impact of Pf parasitemia in PfSPZ-CVac recipients. The period after day 13 was to assess the additional impact of continued chloroquine administration. There was no significant difference in AEs between PfSPZ Vaccine and normal saline (NS) (*P* = 0.215), between PfSPZ-CVac and NS + chloroquine (*P* = 0.822), or between PfSPZ Vaccine and PfSPZ-CVac on days 1–6 (*P* = 0.676) or overall (*P* = 0.128). On days 7–13, more AEs occurred in the subjects receiving PfSPZ-CVac than subjects receiving NS + chloroquine (*P* = 0.073, Barnard’s test, two-sided).

There were no significant differences between solicited AEs in vaccinees and controls (Figure 4) collected 6 days following each immunization. Ten of 56 injections (in nine of 20 subjects receiving 2.7 × 10^6^ PfSPZ of PfSPZ Vaccine) were associated with 17 systemic solicited AEs (five reports of fatigue and three each of arthralgias, headache, myalgias, and subjective fever) compared with one of 18 injections (one report of arthralgias) in the six subjects receiving NS (*P* = 0.215, Barnard’s test two tailed). One event (headache in a vaccine recipient) was grade 2, and all others were grade 1. All events were considered related to IP (Supplemental Table S6). One subject in the PfSPZ Vaccine group experienced one local AE (tenderness) during immunization.

##### PfSPZ-CVac.

There were no significant differences between systemic solicited AEs in vaccinees and controls collected throughout the 70-day immunization period (Figure 4). Seventeen of 55 injections (in 12 of 19 subjects receiving PfSPZ-CVac) were associated with 30 systemic solicited AEs and six local solicited AEs, all grade 2 or less; five of 14 injections (in four of five subjects receiving NS with chloroquine) experienced 10 systemic solicited AEs and no local solicited AEs (*P* = 0.822) (Supplemental Table S6). Seven systemic solicited AEs in five subjects were temporally associated with a positive qPCR for Pf, suggesting these AEs represented symptomatic parasitemia; no solicited systemic AEs were documented in the control subjects during this time period (Figure 4).

#### Comparison of PfSPZ vaccine and PfSPZ-CVac.

Systemic solicited AEs were more frequent after each dose in the PfSPZ-CVac group (occurring in 17 of 55 immunizations) than the PfSPZ Vaccine group (occurring in 10 of 56 immunizations) but were collected over a longer period of time, and the difference was not significant (*P* = 0.128). There was no difference when the first 6 days of solicited AEs for PfSPZ-CVac (occurring in 12 of 55 immunizations) were compared with the first 6 days for PfSPZ Vaccine (occurring in 10 of 56 immunizations) (*P* = 0.676), implying that the excess AEs observed in the PfSPZ-CVac group were associated with the transient parasitemia occurring on day 7–10 or to the continuous use of chloroquine. The role for chloroquine was supported by the comparison of systemic solicited AEs in the two control arms of the study, demonstrating more frequent events in the NS + chloroquine controls (five of 14 injections) than the NS controls (one of 18 injections, *P* = 0.028). Pruritus, a frequently cited adverse effect of chloroquine in African populations, was only noted in two subjects after the first dose with PfSPZ-CVac, was mild, resolved spontaneously, and did not reoccur, despite ongoing chloroquine administration.

#### Laboratory abnormalities following immunization.

There was no significant difference in the number of subjects experiencing laboratory abnormalities between PfSPZ Vaccine and NS control (Supplemental Table S7). All laboratory abnormalities were grade 2 or less. Likewise, there were no differences in vaccinees and controls in the PfSPZ-CVac arms, except that significantly more subjects receiving PfSPZ-CVac experienced grade 1 or 2 neutropenia than controls (*P* = 0.0089) (Supplemental Table S7), although none of the episodes were clinically significant. One subject had an unexplained, unrelated grade 3 elevation of total bilirubin 14 days after the third dose, with all prior and subsequent sample results in the normal range.

#### Unsolicited AEs following immunization.

Nine unsolicited AEs were reported in eight of the 20 subjects receiving PfSPZ Vaccine; one AE was considered probably related to vaccine (acute gastritis, grade 2). One event was considered grade 3 (toothache); all others were grade 2 or less. One AE was reported in controls. Twenty-five unsolicited AEs were reported in 12 of the 19 subjects immunized with PfSPZ-CVac, with five AEs reported in three of the five chloroquine controls; all AEs were grade 2 or less, and none were considered related to IP. The most common unsolicited AEs included toothache, upper respiratory tract infections, and musculoskeletal pain.

#### Serious AEs.

Three serious AEs occurred in three study subjects. An 18-year-old woman was hospitalized for hyperemesis gravidarum. Symptom onset was 19 weeks after her last dose of PfSPZ Vaccine. The remainder of her pregnancy was uneventful, and she delivered a healthy girl at 37 ½ weeks.

A 19-year-old woman was found to have intrauterine fetal demise 9 weeks into her third pregnancy and 9 weeks after her first and only dose of PfSPZ Vaccine. Pregnancy loss before 20 weeks approaches 20% in sub-Saharan Africa^35^; however, the temporal relationship to immunization led the team to consider the event possibly related to vaccine.

A 29-year-old man in the chloroquine + placebo arm of the PfSPZ-CVac group developed non-Hodgkin’s lymphoma. This serious adverse event was considered unrelated to immunization; the details of this case are reported elsewhere (S. Manock et al., manuscript in accepted for publication in AJTMH).

#### *Plasmodium* parasitemia during PfSPZ-CVac immunizations and before CHMI.

**Figure 5.**
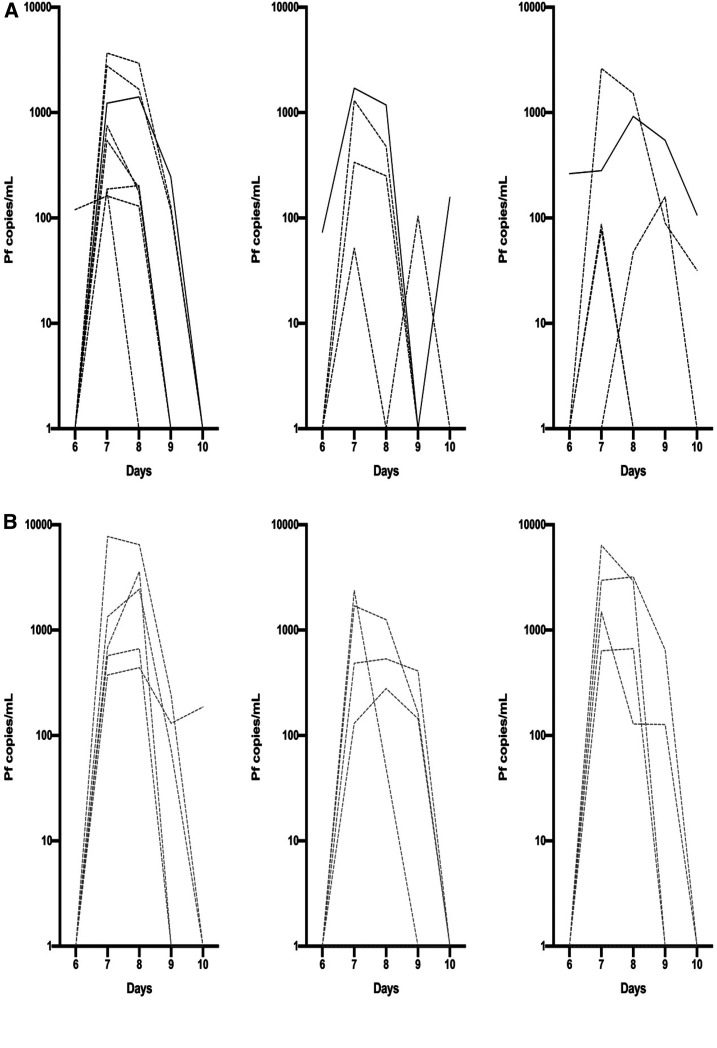
Parasitemia by qPCR after first, second, and third PfSPZ-CVac immunizations. (**A**) Transient parasitemia with each successive dose of PfSPZ Challenge in vaccinees protected after controlled human malaria infection (CHMI). (**B)** Transient parasitemia with each successive dose of PfSPZ Challenge in vaccinees not protected after CHMI. (**C**) Median Pf parasitemia overall and in subjects protected and not protected during CHMI. Only subjects completing all three doses and participating in CHMI (*n* = 13) represented. One subject (#525, solid black line in **A**) was persistently qPCR positive on day 10 after the second and third immunizations and subsequently found to have naturally acquired Pf infection. Two additional subjects positive 10 days after the third (**A**) or first (**B**) dose were qPCR negative at the next measurement 18 days later. This figure appears in color at www.ajtmh.org.

Six to 10 days after each immunization, 17/18 subjects who received three doses of PfSPZ-CVac developed parasitemia by qPCR after the first dose, 13/18 after the second, and third doses (Figure 5). Median parasitemia at each time point was lower in subjects protected during CHMI (Figure 5C). There was no significant correlation between parasitemia with dose 1 and pre-immunization antibody levels to PfCSP (*r*^2^ = 0.10) or PfMSP1 (*r*^2^ = 0.002). There was no significant difference in peak parasitemia between subjects infected and subjects not infected after CHMI (Figure 5C).

During the immunization phase, six subjects had asymptomatic parasitemia outside the 6- to 10-day window during scheduled PCR sampling in two subjects each receiving PfSPZ Vaccine, PfSPZ-CVac, and NS control (Supplemental Table S8). Genotype analysis excluded the PfNF54 strain identified as the cause of parasitemia in three subjects and suggested this was not the etiology in two additional subjects (Supplemental Table S9). Four additional subjects in the PfSPZ-CVac arm (three receiving PfSPZ Challenge and one NS) had asymptomatic Pf parasitemia in the interval between immunization and CHMI. A fifth subject in this group had symptomatic Pf parasitemia with an oral temperature of 38.2°C 14 weeks after third immunization.

#### Adverse events after CHMI.

Solicited AEs were assessed for 5 days after administration of PfSPZ Challenge for CHMI. Controlled human malaria infection was well-tolerated (Supplemental Table S10). One subject in Group 1a had mild pain after injection. One subject in Group 1b had arthralgias 2 days after CHMI which were attributed to physical activity; no other local or systemic AEs were reported during the 5 days after administration of PfSPZ Challenge. Nine unsolicited AEs were reported in eight subjects during the 28 days after CHMI, none of which were deemed related to IP or malaria in blinded assessments.

#### Chloroquine levels during administration of PfSPZ-CVac.

Whole blood chloroquine levels 2 days after the initial loading dose ranged from 15.4 to 129.9 ng/mL; corresponding plasma levels for a subset of samples were uniformly higher than the corresponding whole blood level. All levels were above IC_50_ for the NF54 strain to chloroquine (8.7 ng/mL).^36^

#### Clinical manifestations of malaria after CHMI.

Symptoms or signs of malaria were recorded in 9/21 subjects who developed parasitemia: 4/11, 2/5, and 3/7 who received PfSPZ Vaccine, PfSPZ-CVac, and NS, respectively (Supplemental Table S10). Eight of the nine with symptoms were qPCR-positive and TBS-positive; 1/9 was only qPCR-positive. The median interval between qPCR positivity and symptom onset was 7 days (range, 5–13), and the median interval between TBS positivity and symptom onset was 1 day (range, −1 to +3). All symptoms were mild to moderate; two subjects had elevated temperature (38.2, 38.3°C).

## DISCUSSION

This is the first trial to directly compare the VE of PfSPZ-CVac and PfSPZ Vaccine. At 14–15 weeks after the last dose, a three-dose PfSPZ-CVac regimen of 1.0 × 10^5^ PfSPZ/dose had a VE of 55% (eight of 13, *P* = 0.051), whereas a three-dose PfSPZ Vaccine regimen of 2.7 × 10^6^ PfSPZ/dose had a VE of 27% (five of 15, *P* = 0.32), both against homologous CHMI. The VE of PfSPZ-CVac versus that of PfSPZ Vaccine occurred, despite the fact that 27 times fewer PfSPZ were included in the PfSPZ-CVac regimen. Subjects who received PfSPZ Vaccine and became infected had significantly longer prepatent periods by TBS than did the controls, a finding consistent with previous studies.^18^ Subjects who received PfSPZ-CVac and became infected did not have significantly longer prepatent periods than did controls. We will investigate this unexpected finding in subsequent studies.

During the week after inoculation, the chemo-attenuated PfSPZ in PfSPZ-CVac replicated up to 5 × 10^4^ times in hepatocytes of the vaccinees and expressed ∼3,000 proteins not expressed by the nonreplicating PfSPZ of PfSPZ Vaccine.^13^ Three doses of 9 × 10^5^ PfSPZ of PfSPZ Vaccine protected 72% of vaccinees against homologous CHMI at 9.5 weeks after the last dose in Germany (B. Mordmüller, personal communication) and 64% of vaccinees in the United States against homologous CHMI at ∼19 weeks after the last vaccine dose,^12^ and three doses of 5 × 10^4^ PfSPZ of PfSPZ-CVac protected 100% of vaccinees in Germany against homologous CHMI at 10 weeks after the last dose.^13^ Thus, the better VE with a much lower dose of PfSPZ-CVac in our trial was expected.

Data from mice and nonhuman primates indicate the VE of radiation-attenuated and chemo-attenuated PfSPZ is dependent on CD8^+^ T cells; antibodies alone are not adequate.^7,37–39^ Nonetheless, in a previous study of PfSPZ Vaccine, protected vaccinees had significantly higher levels of antibodies to PfCSP than unprotected vaccinees.^8^ In this study, at the time of CHMI, uninfected PfSPZ Vaccine vaccinees had 2.9 times higher levels of antibodies to PfCSP than did infected vaccinees (2936 versus 1012, *P* = 0.031), and uninfected PfSPZ-CVac vaccinees (618) had 2.7 times higher levels of antibodies to PfCSP than did infected vaccinees (293) (*P* = 0.126). Most strikingly, infected (non-protected) PfSPZ Vaccine vaccinees had significantly higher levels of antibodies than uninfected (protected) PfSPZ-CVac vaccinees. Within a particular group (e.g., PfSPZ Vaccine or PfSPZ-CVac), there was an association between levels of antibodies to PfCSP and protection, but between groups, this was not the case. Within a group, levels of antibodies to PfCSP are a biomarker for protection and may contribute marginally to protection; on the other hand, the complete lack of association when antibodies are compared between PfSPZ Vaccine and PfSPZ-CVac is consistent with CD8^+^ T cells being the primary mediator of protection.

Conducting this second ever clinical trial of an IP in Equatorial Guinea introduced challenges and potential limitations to the interpretation of the results. Serious AEs that led to halting of the trial disrupted the schedule for immunizations and CHMI in some of the subjects, leading to differences in the time between the second and third doses and the intervals between the third dose and CHMI. Naturally acquired, asymptomatic malaria was discovered in some of the subjects before undergoing CHMI, and this had to be treated, which led to a delay in the CHMIs or, in some cases, the subjects not undergoing CHMI. The investigative team was also challenged by several unrelated SAEs that required substantial amounts of their time (e.g., lymphoma in a young adult).

One of the seven control subjects who participated in CHMI did not develop Pf parasitemia by either TBS or qPCR. This substantially contributed to lower VE calculations in both arms of the study. Although 100% of nonimmune control subjects in the United States and Europe (73/73)^13,26,27,29,40,41^ and Tanzania (34/34)^15,18^ have been infected with this dose, in other settings in Africa, including Gabon (20/25),^27,42^ Gambia (17/19),^28^ and unpublished data from Mali (8/15) and Kenya (137/170) (submitted), this has not been the case.

The doses of PfSPZ Vaccine and PfSPZ-CVac were probably not optimal, based on the results of concurrent studies in similar populations. In studies of the VE against homologous CHMI of PfSPZ Vaccine in Tanzania, it has been shown that three immunizations of 9.0 × 10^5^ PfSPZ administered at 8-week intervals resulted in VE of 100% against CHMI conducted 3–11 weeks after the third immunization. However, when the dose was increased to 1.8 × 10^6^ PfSPZ administered at 8-week intervals, VE against CHMI conducted 7 weeks after the third immunization was reduced to 33%, suggesting doses can be too high.^18^ When the EGSPZV2 trial was designed, it was thought that higher doses would be better, thus the choice of 2.7 × 10^6^ PfSPZ per dose. The resulting VE of 27% is consistent with the findings in Tanzania, increasing the dose beyond 9.0 × 10^5^ apparently reduces VE against CHMI. Thus, in our next studies, we plan to immunize with 9.0 × 10^5^ PfSPZ.

The dose of PfSPZ-CVac, 1.0 × 10^5^ PfSPZ, was two times higher than the dose that achieved 100% VE in malaria-naive adults in Tubingen, Germany.^13^ A regimen of three doses of 2.0 × 10^5^ PfSPZ at 1-month intervals was tested in Mali for protection against naturally transmitted malaria and gave suboptimal results (Thera and Laurens, unpublished). The results indicated that higher doses of PfSPZ-CVac are needed in Africa because of naturally acquired immunity and an associated immune hyporesponsiveness to malaria immunogens. Immune hyporesponsiveness could also explain the limited decrease in parasitemia seen after each successive immunization in this study, unlike PfSPZ-CVac trials in malaria-naive adults, where a consistent decrease in mean parasitemia is seen in all subjects with each successive dose.^13,43^ A subsequent study in Mali assessing the VE of PfSPZ-CVac against naturally transmitted malaria is using three doses of 4.0 × 10^5^ PfSPZ (NCT03952650).

For subjects immunized with radiation-attenuated PfSPZ Vaccine, there were no significant differences in the number of solicited systemic AEs between PfSPZ Vaccine and NS recipients. Likewise, there was no significant difference between vaccinees and controls in the first 5 days after each dose of PfSPZ-CVac. However, solicited AEs were more frequent in vaccinees receiving PfSPZ-CVac than controls during days 7–13 after each immunization (*P* = 0.073), which we attribute to symptoms related to parasitemia in the 6–12 days after immunization. In addition, AEs attributed to chloroquine accounted for 1/3 of the AEs reported in vaccinees and controls in the PfSPZ-CVac group and account for the differences in the numbers of AEs between the NS + chlorquine control group and the NS control group. Despite parasitemia-associated AEs occurring exclusively in vaccinees, there was no significant difference in the number of solicited systemic AEs between those receiving PfSPZ-CVac (PfSPZ Challenge + chloroquine) and those receiving NS and chloroquine. It was noteworthy that only two of 19 individuals experienced pruritus during 9 weeks of chloroquine administration; in both cases, it was mild, resolved spontaneously, and did not reoccur, despite ongoing chloroquine dosing.

In summary, as part of an ongoing effort to optimize the dosage regimens for PfSPZ Vaccine and PfSPZ-CVac, we conducted a trial comparing a single dosage regimen of each. *Plasmodium falciparum* sporozoites-CVac provided higher VE than did PfSPZ Vaccine at a much lower dose. However, neither regimen was optimal. Higher doses of PfSPZ-CVac and lower doses of PfSPZ Vaccine will be assessed next.

## Supplemental appendix, tables, and figures

Supplemental materials
