# Supplementary material for: Immunogenicity and Protective Efficacy of Radiation-Attenuated and Chemo-Attenuated PfSPZ Vaccines in Equatoguinean Adults
Source: Am J Trop Med Hyg. 2020 Nov 16;104(1):283–93. doi: 10.4269/ajtmh.20-0435 (PMC7790068; doi:10.4269/ajtmh.20-0435)
Supplement: Supplementary file 1 [file tpmd200435.SD1.pdf]

SUPPLEMENTAL DATA

Figure legends

Figure S1. Proposed study schedule and schedule as executed. This trial was originally designed to enroll 26 subjects into the PfSPZ Vaccine arm and 26 subjects into the PfSPZ-CVac arm and randomize within each arm 20 subjects to vaccine and 6 subjects to control. Immunizations in the PfSPZ-Vaccine arm were scheduled for 1, 9 and 17 weeks and PfSPZ-CVac for 9, 13 and 17 weeks so that both groups could undergo CHMI at the same time (10±1 weeks after the 3rd dose). Due to challenges in recruitment the PfSPZ-CVac arm was broken into 2 cohorts, the first of which began immunizations on schedule with the second cohort delayed by 5 weeks. An unanticipated safety hold to evaluate a SAE led to additional delay with the 3rd dose for the second cohort administer 13 weeks after the second dose instead of 4 weeks. For these subjects CHMI was delayed to allow a minimum of 10 weeks between the 3rd dose and CHMI. A few subjects encountered additional dealys due to intercurrent malaria infections from natural exposure.

Figure S2. Antibodies to PfCSP and MSP-1 by ELISA. IgG antibodies to Pf circumsporozoite protein PfCSP by ELISA two weeks after the 3rd dose (panel A) and at the time of CHMI (panel B) comparing PfSPZ Vaccine and PfSPZ-CVac are presented as OD 1.0 ratios and correspond to the net OD 1.0 values presented in Figure 3 panels A and B. IgG antibodies to Pf circumsporozoite protein PfCSP by ELISA two weeks after the 3rd dose (panel C) and at the time of CHMI (panel D) comparing infected and uninfected subjects in PfSPZ Vaccine and PfSPZ-CVac are presented as OD 1.0 ratios and correspond to the net OD 1.0 values presented in Figure 3 panels C and D. IgG antibodies to Pf merozoite surface protein-1 PfPfMSP-1 by ELISA

measured at the time of CHMI (panel E) comparing PfSPZ Vaccine and PfSPZ-CVac. Filled circles (●) represent subjects remaining uninfected after CHMI; open circles (○) represent subjects infected after CHMI.

Antibody responses to PfCSP 2 weeks after the 3rd dose (panel A) were significantly higher in the PfSPZ Vaccine group (median OD 1.0 ratio = 38.70) than in the PfSPZ-CVac group (median OD 1.0 ratio = 2.48) (p=0.0043, Wilcoxon signed-rank test, 2 tailed). The PfSPZ-CVac group had higher antibody levels than normal saline controls 2 weeks after 3rd dose (median OD 1.0 ratio = 1.02) (p<0.0001, Wilcoxon signed-rank test, 2 tailed). Antibody responses to PfCSP the day prior to CHMI (panel B) were significantly higher in the PfSPZ Vaccine group (median OD 1.0 ratio 43.84) than in the PfSPZ-CVac group (OD 1.0 ratio 4.10) (p<0.0001, Mann-Whitney test, 2 tailed). The PfSPZ-CVac group had higher antibody levels than normal saline controls prior to CHMI (median OD 1.0 ratio = 1.27, p<0.0001, Wilcoxon signed-rank test, 2 tailed).

Median OD 1.0 ratio of PfCSP antibodies measured 2 weeks after the 3rd dose (panel C) in the PfSPZ Vaccine group were higher in uninfected vs that in infected subjects (median OD 1.0 ratio 67.57 vs 40.35, p=0.59, Wilcoxon signed-rank test, 2 tailed), but the difference was not significant. Likewise, there was no significant difference in antibody levels 2 weeks after the 3rd dose between subjects who received PfSPZ-CVac who were not infected, versus those who became infected (median OD 1.0 ratio 3.76 vs 4.90, p=0.93).

Prior to CHMI (panel D) the uninfected PfSPZ Vaccine group also had a higher median OD 1.0 ratio, but this did not reach the level of statistical significance (median OD 1.0 ratio 61.28 vs

20.11, p=0.15, Wilcoxon signed-rank test, 2 tailed). In subjects who received PfSPZ-CVac who were uninfected or infected the median OD 1.0 ratios was higher, but not significantly (6.04 vs 3.49, p=0.35).

In subjects who received PfSPZ Vaccine who were uninfected the PfMSP-1 (panel E) median OD 1.0 measured prior to CHMI was higher than that of infected subjects (median OD 1.0 = 889 vs 62), but not significantly (p=0.406) (Table S5). Subjects who received PfSPZ-CVac and were uninfected also had higher antibodies to PfMSP-1 prior to CHMI than the infected subjects (median OD 1.0 = 1518 vs 605), but the difference was not significant (p=0.880) (Table S5).

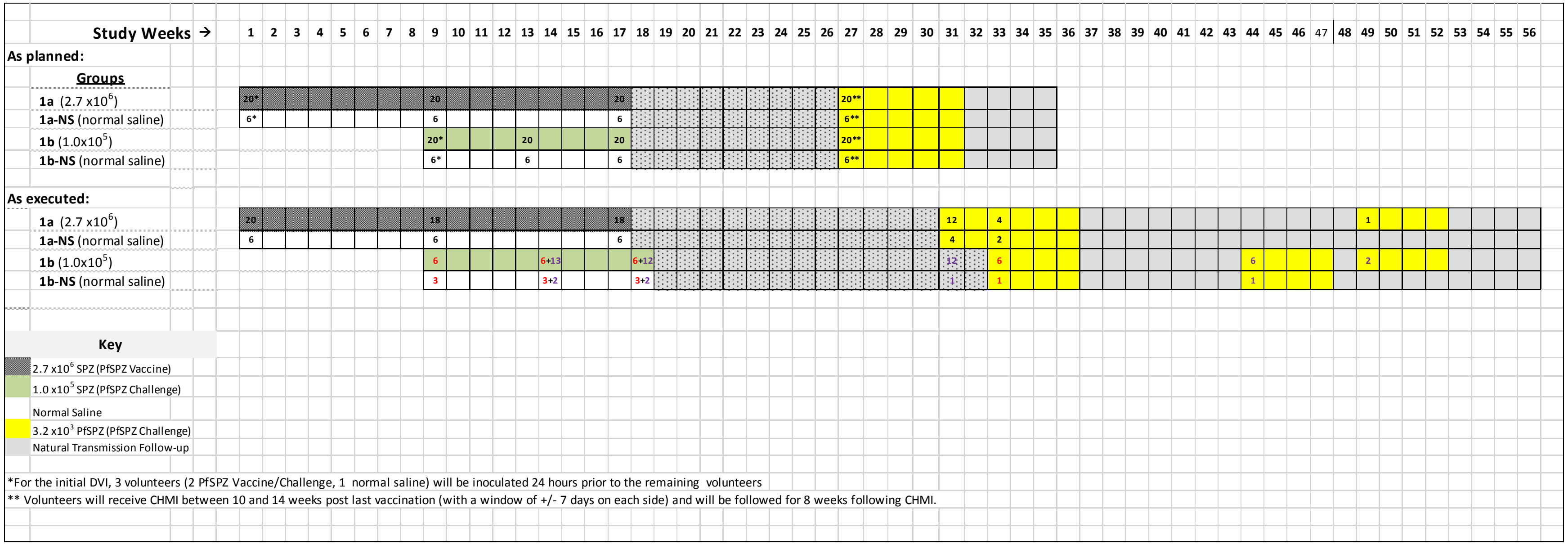
Study Weeks →
As planned:
Groups
1a (2.7 x10^6)
1a-NS (normal saline)
1b (1.0x10^5)
1b-NS (normal saline)
As executed:
1a (2.7 x10^6)
1a-NS (normal saline)
1b (1.0x10^5)
1b-NS (normal saline)
Key
2.7 x10^6 SPZ (PfSPZ Vaccine)
1.0 x10^5 SPZ (PfSPZ Challenge)
Normal Saline
3.2 x10^3 PfSPZ (PfSPZ Challenge)
Natural Transmission Follow-up
*For the initial DVI, 3 volunteers (2 PfSPZ Vaccine/Challenge, 1 normal saline) will be inoculated 24 hours prior to the remaining volunteers
** Volunteers will receive CHMI between 10 and 14 weeks post last vaccination (with a window of +/- 7 days on each side) and will be followed for 8 weeks following CHMI.

Figure S1.

A. 2 weeks after 3rd dose.

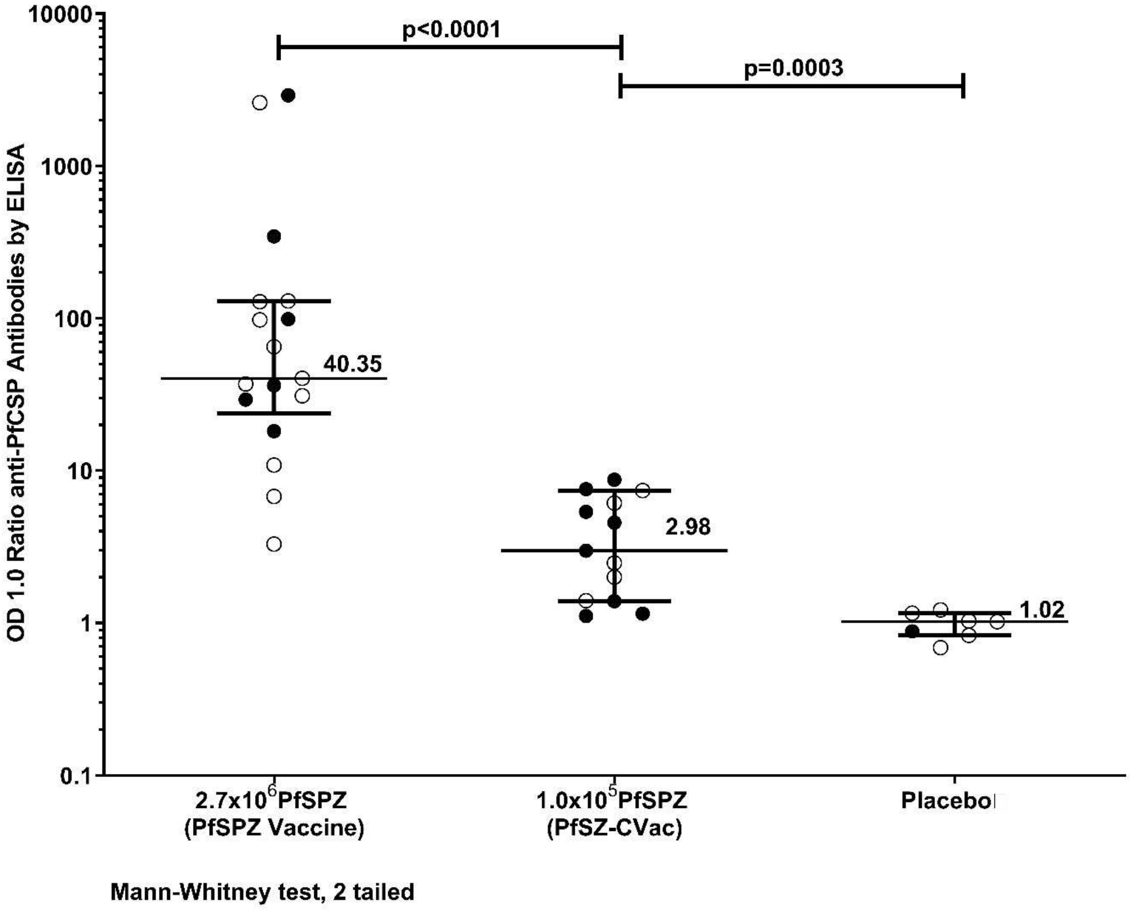

B. Pre-CHMI

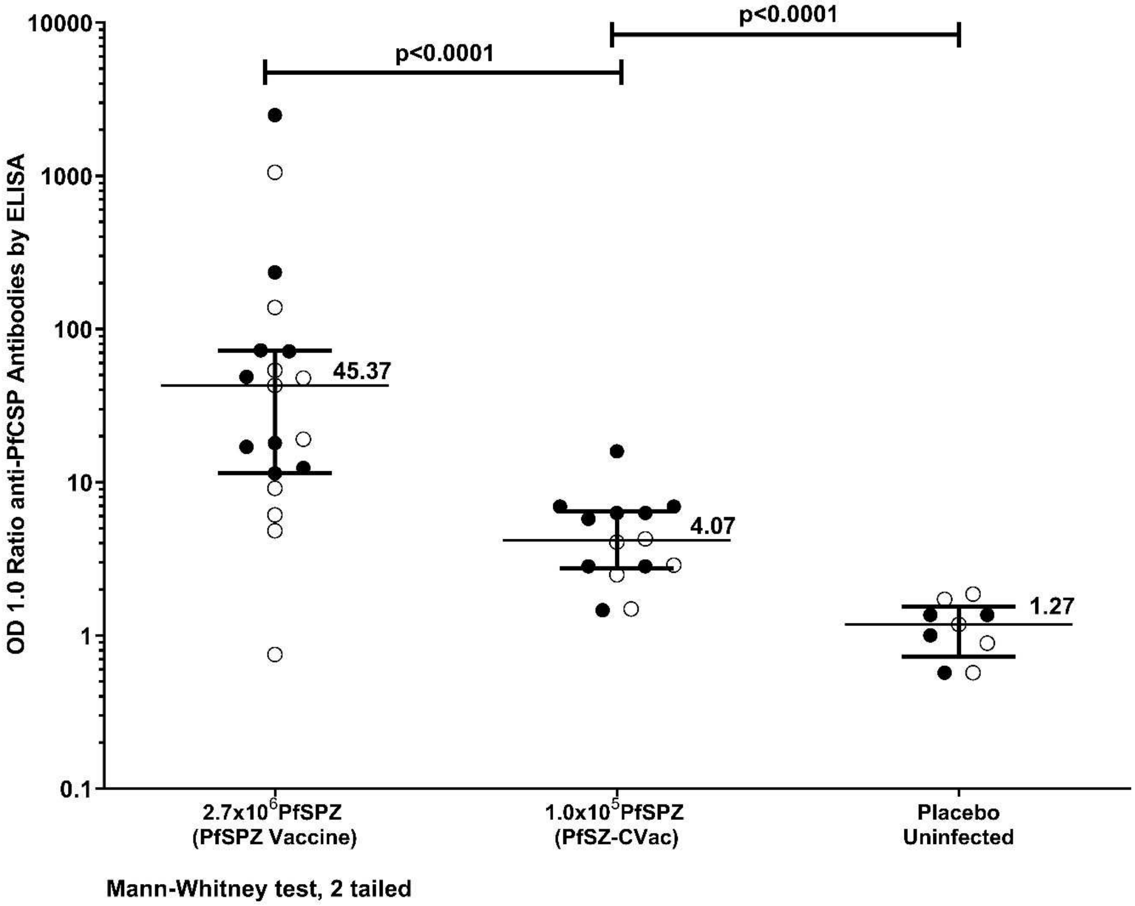

p<0.0001
p<0.0001
10000
1000
100
10
1
0.1
OD 1.0 Ratio anti-PfCSP Antibodies by ELISA
45.37
4.07
1.27
2.7x10^6 PfSPZ
(PfSPZ Vaccine)
1.0x10^5 PfSPZ
(PfSZ-CVac)
Placebo
Uninfected
Mann-Whitney test, 2 tailed

C. 2 weeks after 3rd dose

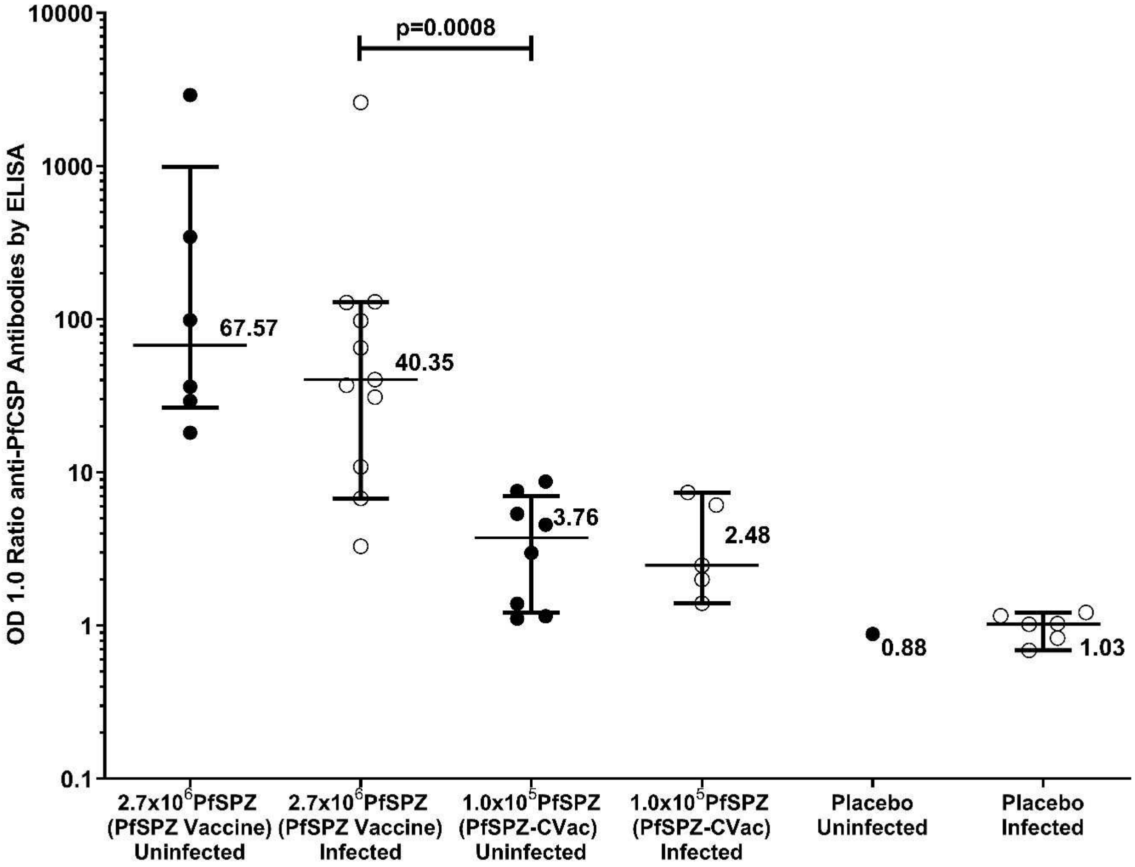

D. Pre-CHMI

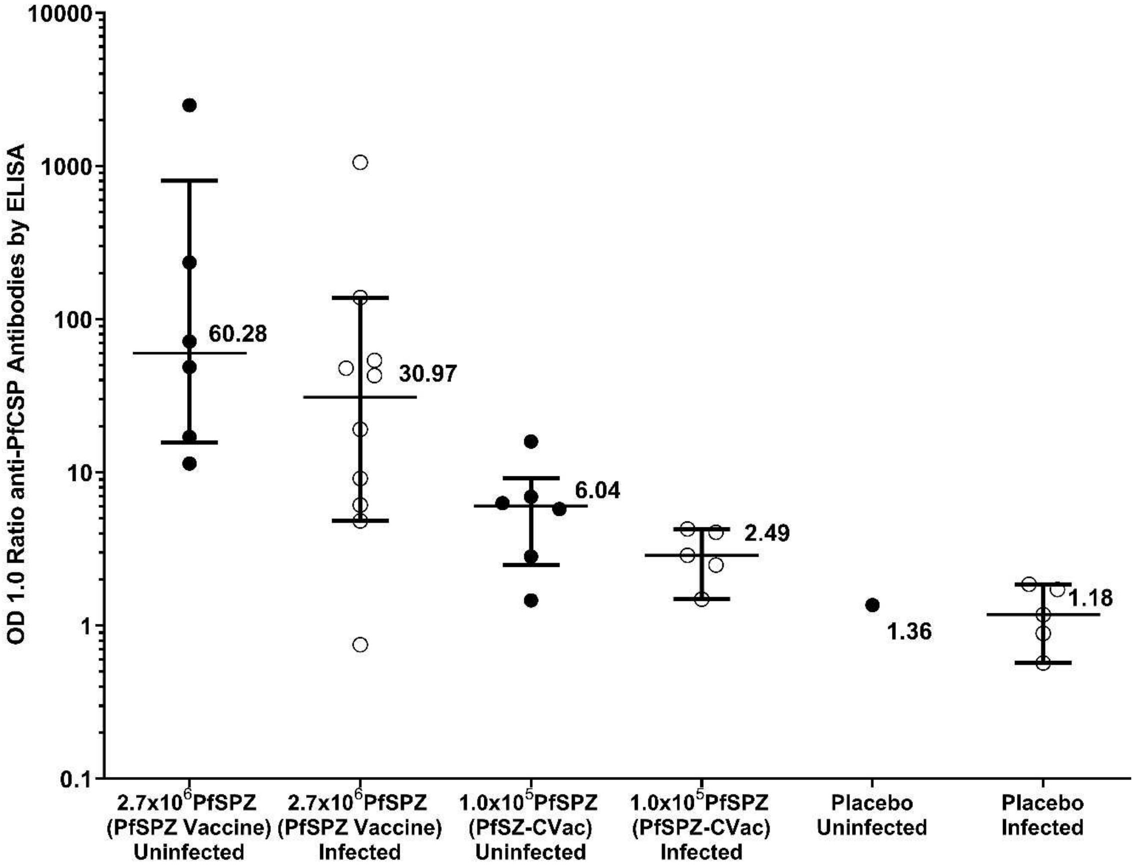
OD 1.0 Ratio anti-PfCSP Antibodies by ELISA
10000
1000
100
10
1
0.1
60.28
30.97
6.04
2.49
1.36
1.18
2.7x10^6 PfSPZ (PfSPZ Vaccine) Uninfected
2.7x10^6 PfSPZ (PfSPZ Vaccine) Infected
1.0x10^5 PfSPZ (PfSZ-CVac) Uninfected
1.0x10^5 PfSPZ (PfSPZ-CVac) Infected
Placebo Uninfected
Placebo Infected

E. PfMSP-1 pre-CHMI

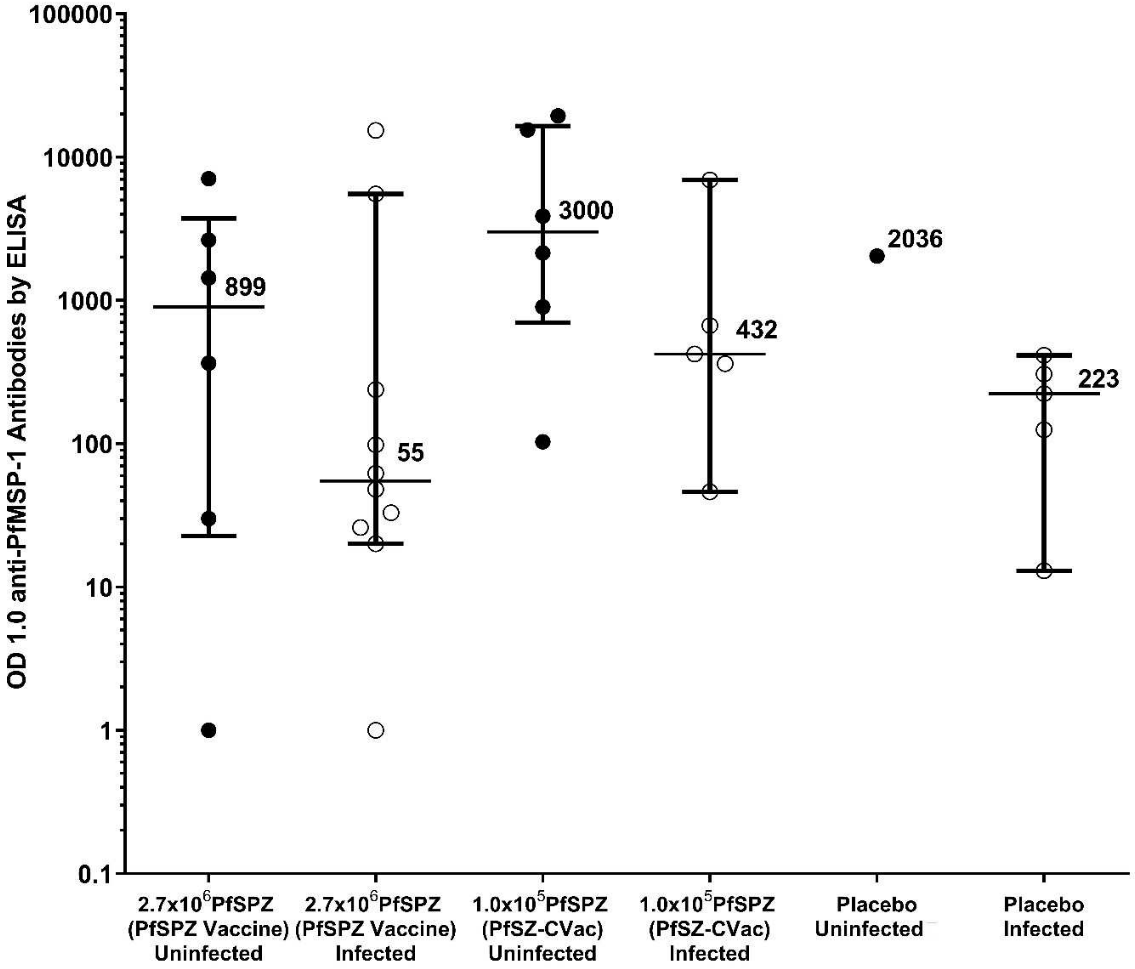

Figure S2

Table S1: Inclusion Criteria

1. Healthy males and females, based on clinical and laboratory findings
2. Age 6 months to 65 years
3. Adults with a Body Mass Index (BMI) 18 to 30 Kg/m^2; or adolescents, children and infants with Z-score of the selected indicator ([weight-for-height], [(height and BMI) for age]) category within ±2SD.
4. Long-term (at least one year) or permanent residence in the Baney district or Malabo city
5. Agreement to release medical information and to inform the study doctor concerning contraindications for participation in the study
6. Willingness to be attended to by a study clinician and take all necessary medications prescribed during study period
7. Agreement to provide contact information of a third party household member or close friend to study team
8. Agreement not to participate in another clinical trial during the study period
9. Agreement not to donate blood during the study period
10. Able and willing to complete the study visit schedule over the study follow up period, including the hospitalizations required for protocol compliance
11. Willingness to undergo HIV, hepatitis B (HBV) and hepatitis C (HCV) tests
12. Volunteer (subjects 18 years of age and older) or the parent / guardian signing the informed consent (for subjects <18 years of age) is able to demonstrate their understanding of the study by responding correctly to 10 out of 10 true/false statements (in a maximum of two attempts for those who failed to respond correctly to all true/false statements in the first attempt).
13. Signed written informed consent, in accordance with local practice, provided by adult volunteers, parents or legal representatives and relevant assent for children participants as applicable.
14. Free from malaria parasitemia by blood smear at enrollment and by PCR for group 1
15. Has not been treated with any antimalarial medication for at least two weeks prior to the first immunization.
16. Free from helminth infections (detected by microscopy) at enrollment.
17. Female volunteers aged 9 years and above must be non-pregnant (as demonstrated by a negative urine pregnancy test), and those aged 13 to 49 years provide consent/assent of their willingness to take protocol-defined measures not to become pregnant during the study and safety follow-up period.

Table S2: Exclusion Criteria

1. Previous receipt of an investigational malaria vaccine in the last 5 years
2. Participation in any other clinical study involving investigational medicinal products including investigational malaria drugs within 30 days prior to the onset of the study or during the study period
3. History of arrhythmias or prolonged QT-interval or other cardiac disease, or clinically significant abnormalities in electrocardiogram (ECG) at screening
4. Positive family history in a 1st or 2nd degree relative for cardiac disease at age <50 years old
5. A history of psychiatric disease
6. Suffering from any chronic illness including; diabetes mellitus, cancer or HIV/AIDS
7. Any confirmed or suspected immunosuppressive or immune-deficient condition, including asplenia
8. History of drug or alcohol abuse interfering with normal social function
9. The use of chronic immunosuppressive drugs or other immune modifying drugs within three months of study onset (inhaled and topical corticosteroids are allowed) and during the study period
10. Any clinically significant deviation from the normal range in biochemistry or hematology blood tests or in urine analysis
11. Positive HIV, hepatitis B virus or hepatitis C virus tests
12. Volunteers who are have risk factors for tuberculosis and/or signs and symptoms of tuberculosis (TB), plus a positive tuberculin skin test (TST).
13. Symptoms, physical signs and laboratory values suggestive of systemic disorders including renal, hepatic, blood, cardiovascular, pulmonary, skin, immunodeficiency, psychiatric, and other conditions which could interfere with the interpretation of the study results or compromise the health of the volunteers
14. Any medical, social condition, or occupational reason that, in the judgment of the investigator, is a contraindication to protocol participation or impairs the volunteer’s ability to give informed consent, increases the risk to the volunteer because of participation in the study, affects the ability of the volunteer to participate in the study or impairs the quality, consistency or interpretation of the study data.
15. History of non-febrile seizures or atypical febrile seizures.

Table S3: List of solicited adverse events with the grading system for severity and grading for relatedness*.

Local Solicited AEs *(at injection site)*		• Pain<br>• Tenderness<br>• Pruritus	1<br>2<br>3	Daily activity minimally affected, with or without treatment<br>Daily activity possible but only with treatment<br>Daily activity not possible even with treatment
		• Erythema<br>• Swelling<br>• Induration<br>• Bruising/extravasated blood	1<br>2<br>3	2.5 – 5 cm<br>5.1 – 10 cm<br>>10 cm, necrosis or exfoliative dermatitis
Systemic Solicited (Core List-post Vaccination)		• Fever	1<br>2<br>3	38.0°C – 38.4°C<br>38.5°C – 38.9°C<br>>39.0°C
	Adults, adolescents and older children	• Allergic reaction *(rash, urticaria, pruritis, edema)*<br>• Headache<br>• Subjective Fever**<br>• Fatigue<br>• Malaise<br>• Chills<br>• Myalgia<br>• Arthralgia	1<br>2<br>3	Daily activity minimally affected, with or without treatment<br>Daily activity possible but only with treatment<br>Daily activity not possible even with treatment
	Infants and younger children	• Allergic reaction *(rash, urticaria, pruritis, edema)*<br>• Subjective fever*<br>• Drowsiness<br>• Irritability/fussiness<br>• Inability/refusal to eat or drink		
Post CHMI Malaria Signs and Symptoms (In addition to Core List)		• Dizziness<br>• Rigors<br>• Sweats<br>• Cough<br>• Nausea<br>• Vomiting<br>• Abdominal pain<br>• Diarrhea<br>• Chest pain<br>• Palpitations<br>• Shortness of breath	1<br>2<br>3	Daily activity minimally affected, with or without treatment<br>Daily activity possible but only with treatment<br>Daily activity not possible even with treatment

*AEs (solicited and unsolicited) were recorded and graded by physicians: mild (easily tolerated), moderate (interfere with normal activity), severe (prevents normal activity) or life threatening (Table S3). Axillary temperature was Grade 1 (38.0-38.4°C), Grade 2 (38.5–38.9°C) or Grade 3 (> 39.0°C). Hematological and biochemical abnormalities were assessed using standard clinical assays. All AEs were assessed for severity and relatedness to IP administration. AEs were classified as definitely related, probably related, possibly related, unlikely to be related, or not related. Definitely, probably, and possibly were classified as related to IP administration; unlikely to be related and not related were classified unrelated.

*** Perceived by the subject and/or subject's guardian*

Table S4: Solcited adverse events for CVac

Chloroquine Only Dosing Period	Post Immunization during CQ Administration	
CQ	Chloroquine + PfSPZ Challenge (CQ+CH)	Chloroquine + Parasitemia (CQ+ P)
CQ solicited AEs will be collected from the Day of the first dose through +7 days after the last dose.	*Six (6) additional signs/symptoms (with CQ solicited AEs) will be solicited from day of PfSPZ Challenge Vaccination through +5 days.*	*Twelve (12) additional signs/symptoms (with CQ solicited AEs) will be solicited from +6 days following PfSPZ Challenge Vaccination through +12 days.*
**Days CV-2, CV-1**<br>**Days 14 to 28 (CV_1+13 to CV_1+27)**<br>**Days 42 to 56 (CV_2+13 to CV_2+27)**	**Days 1 to 6 (CV_1 to CV_1+5)**<br>**Days 29 to 34 (CV_2 to CV_2+5)**<br>**Days 57 to 62 (CV_3 to CV_3+5)**	**Days 7 to 13 (CV_1+6 to CV_1+12)**<br>**Days 35 to 41 (CV_2+6 to CV_2+12)**<br>**Days 63 to 69 (CV_3+6 to CV_3+12)**
1 Nausea	1 Nausea	1 Nausea
2 Vomiting	2 Vomiting	2 Vomiting
3 Diarrhea	3 Diarrhea	3 Diarrhea
4 Abdominal pain	4 Abdominal pain	4 Abdominal pain
5 Dizziness	5 Dizziness	5 Dizziness
6 Tinnitus	6 Tinnitus	6 Tinnitus
7 Blurred vision	7 Blurred vision	7 Blurred vision
8 Photosensitivity	8 Photosensitivity	8 Photosensitivity
9 Insomnia	9 Insomnia	9 Insomnia
10 Pruritus	10 Pruritus	10 Pruritus
11 Headache	11 Headache	11 Headache
12 Fatigue	12 Fatigue	12 Fatigue
13 Myalgia	13 Myalgia	13 Myalgia
14 Anxiety	14 Anxiety	14 Anxiety
15 Confusion	15 Confusion	15 Confusion
	16 Elevated body temperature of >38oC	16 Elevated body temperature of >38oC
	17 Allergic reaction (rash, urticaria, pruritus, edema)	17 Allergic reaction (rash, urticaria, pruritus, edema)
	18 Subjective fever	18 Subjective fever
	19 Malaise	19 Malaise
	20 Chills	20 Chills
	21 Arthralgia	21 Arthralgia
		20 Rigors
		21 Sweats
		22 Cough
		23 Chest pain
		24 Palpitations
		25 Shortness of breath

CV= PfSPZ Challenge Vaccination

Table S5. Antibodies to PfCSP and PfMSP1. All out-of-range, negative and zero values are reported as 1.

Group (Age)	PfSPZ/ Dose	Infection	Volunteer ID	ELISA PfCSP OD 1.0							ELISA PfMSP-1 OD 1.0	
				Pre-Immune	2 weeks post-3rd dose	NET (Pre-Post)	Ratio (Post/Pre)	pre-CHMI	NET pre-CHMI	Ratio (Post/Pre)	Pre-Immune	pre-CHMI
1a (18-35 y)	2.7x10[6] PfSPZ Vaccine	Uninfected	E21A317	30	2,966	2,936	98.87	2,182	2,152	71.73	1	1
			E21A371	198	5,797	5,599	29.28	3,575	3,377	17.06	2,283	2,632
			E21A412	130	2,358	2,228	18.14	1,615	1,485	11.42	8,505	7,052
			E21A414	319	11,569	11,250	36.27	15,892	15,573	48.82	355	365
			E21A416	-	2,911	2,911	2,911.00	2,494	2,494	2,494.00	-	1,432
			E21A444	17	5,867	5,850	345.12	4,014	3,997	235.12	1	30
			**Median**	**130**	**4,382**	**4,268**	**67.57**	**3,035**	**2,936**	**60.28**	**355**	**899**
		Infected	E21A309	39	5,021	4,982	128.74	5,442	5,403	138.54	48	33
			E21A311	108	4,001	3,893	37.05	1,094	986	9.13	121	98
			E21A313	224	14,587	14,363	65.12	4,504	4,280	19.11	67	48
			E21A314	73	2,261	2,188	30.97	520	447	6.12	19	20
			E21A316	21	2,052	2,031	97.71	1,027	1,006	47.90	28	26
			E21A399	1	2,601	2,600	2,601.00	1,057	1,056	1,056.00	24,988	15,331
			E21A402	55	599	544	10.89	320	265	4.82	447	238
			E21A417	1,363	4,499	3,136	3.30	2,381	1,018	0.75	11,376	5,534
			E21A426	958	6,474	5,516	6.76	-	-	-	-	-
			E21A433	31	1,251	1,220	40.35	1,359	1,328	42.84	1	1
			E21A448	7	909	902	129.86	384	377	53.86	83	62
			**Median**	**55**	**2,601**	**2,600**	**40.35**	**1,076**	**1,012**	**30.97**	**75**	**55**
		**Group Median**		**64**	**2,966**	**2,936**	**40.35**	**1,899**	**1,407**	**45.37**	**83**	**80**
1b (18-35 y)	1.0x10[5] PfSPZ-CVac	Uninfected	E21B-407	98	740	642	7.55	618	520	6.31	20,612	19,348
			E21B-446	104	906	802	8.71	722	618	6.94	50	103
			E21B-508	337	386	49	1.15	954	617	2.83	4,743	3,864
			E21B-509	359	1,633	1,274	4.55	5,721	5,362	15.94	1,021	2,136
			E21B-518	464	643	179	1.39	677	213	1.46	13,377	15,448
			E21B-525	284	316	32	1.11	-	-	-	-	-
			E21B-526	247	1,327	1,080	5.37	1,425	1,178	5.77	234	899
			E21B-530	52	155	103	2.98	-	-	-	-	-
			**Median**	**266**	**692**	**411**	**3.76**	**838**	**618**	**6.04**	**2,882**	**3,000**
		Infected	E21B-379	210	1,555	1,345	7.40	898	688	4.28	1	423
			E21B-401	139	345	206	2.48	566	427	4.07	1,377	669
			E21B-458	112	685	573	6.12	323	211	2.88	32	361
			E21B-519	196	275	79	1.40	489	293	2.49	5,919	6,908
			E21B-527	257	515	258	2.00	384	127	1.49	88	46
			**Median**	**196**	**515**	**258**	**2.48**	**489**	**293**	**2.49**	**88**	**423**
		**Group Median**		**210**	**643**	**258**	**2.98**	**677**	**520**	**4.07**	**1,021**	**899**

**1b (18-35 y)**	**Placebo**	**UnInfected**	E21A422	108	95	1	0.88	147	39	1.36	1,525	2,036
		**Infected**	E21A411	979	814	1	0.83	559	1	0.57	547	223
			E21A303	64	44	1	0.69	57	1	0.89	1	13
			E21A431	254	294	40	1.16	300	46	1.18	191	306
			E21A472	18	22	4	1.22	31	13	1.72	303	414
			E21B-353	92	95	3	1.03	171	79	1.86	97	125
			E21B-459	48	49	1	1.02	-	-	-	-	-
	**Group Median**			**92**	**95**	**1**	**1.02**	**159**	**26**	**1.27**	**247**	**265**

Table S6: Solicited Adverse Events Post-Vaccination. Adverse events are shown as the number of subjects (% of subjects) experiencing the adverse event by dose and stratified according to the greatest severity reported. Boxes are shaded to highlight the positive responses (blue – no grade assigned; yellow – mild; orange – moderate). Gray shaded boxes represent symptoms not solicited for PfSPZ Vaccine.

		PfSPZ Vaccine						PfSPZ-CVac					
		2.7x10^{6}			Placebo			1.0x10^{5}			Placebo		
**Solicited Event**	**Grade**	**Dose 1 N=20**	**Dose 2 N=18**	**Dose 3 N=18**	**Dose 1 N=6**	**Dose 2 N=6**	**Dose 3 N=6**	**Dose 1 N=19**	**Dose 2 N=18**	**Dose 3 N=18**	**Dose 1 N=5**	**Dose 2 N=5**	**Dose 3 N=4**
**Local Adverse Events**													
Bruising	Grade 1-Mild	0	0	0	0	0	0	0	0	0	0	0	0
	Grade 2-Moderate	0	0	0	0	0	0	0	0	0	0	0	0
	Grade 3-Severe	0	0	0	0	0	0	0	0	0	0	0	0
Erythema	Grade 1-Mild	0	0	0	0	0	0	0	0	0	0	0	0
	Grade 2-Moderate	0	0	0	0	0	0	0	0	0	0	0	0
	Grade 3-Severe	0	0	0	0	0	0	0	0	0	0	0	0
Induration	Grade 1-Mild	0	0	0	0	0	0	0	0	0	0	0	0
	Grade 2-Moderate	0	0	0	0	0	0	0	0	0	0	0	0
	Grade 3-Severe	0	0	0	0	0	0	0	0	0	0	0	0
Pain	Grade 1-Mild	0	0	0	0	0	0	1 (5.3)	1 (5.6)	1 (5.6)	0	0	0
	Grade 2-Moderate	0	0	0	0	0	0	0	0	0	0	0	0
	Grade 3-Severe	0	0	0	0	0	0	0	0	0	0	0	0
Pruritus	Grade 1-Mild	0	0	0	0	0	0	0	0	0	0	0	0
	Grade 2-Moderate	0	0	0	0	0	0	0	0	0	0	0	0
	Grade 3-Severe	0	0	0	0	0	0	0	0	0	0	0	0
Swelling	Grade 1-Mild	0	0	0	0	0	0	0	0	0	0	0	0
	Grade 2-Moderate	0	0	0	0	0	0	0	0	0	0	0	0
	Grade 3-Severe	0	0	0	0	0	0	0	0	0	0	0	0
Tenderness	Reported*	1 (5.0)	0	0	0	0	0	0	0	0	0	0	0
	Grade 1-Mild	0	0	0	0	0	0	1 (5.3)	1 (5.6)	1 (5.6)	0	0	0
	Grade 2-Moderate	0	0	0	0	0	0	0	0	0	0	0	0

		PfSPZ Vaccine						PfSPZ-CVac					
		2.7x10^6			Placebo			1.0x10^5			Placebo		
**Solicited Event**	**Grade**	**Dose 1 N=20**	**Dose 2 N=18**	**Dose 3 N=18**	**Dose 1 N=6**	**Dose 2 N=6**	**Dose 3 N=6**	**Dose 1 N=19**	**Dose 2 N=18**	**Dose 3 N=18**	**Dose 1 N=5**	**Dose 2 N=5**	**Dose 3 N=4**
	Grade 3-Severe	0	0	0	0	0	0	0	0	0	0	0	0
**Systemic Adverse Events**													
Abdominal Pain	Grade 1-Mild							0	1 (5.6)	1 (5.6)	0	0	0
	Grade 2-Moderate							0	0	1 (5.6)	0	0	0
	Grade 3-Severe							0	0	0	0	0	0
Anxiety	Grade 1-Mild							0	0	0	0	0	0
	Grade 2-Moderate							0	0	0	0	0	0
	Grade 3-Severe							0	0	0	0	0	0
Arthralgia	Reported*	1 (5.0)	0	0	0	0	0	0	0	0	0	0	0
	Grade 1-Mild	0	1 (5.6)	1 (5.6)	0	1 (16.7)	0	0	0	0	0	0	0
	Grade 2-Moderate	0	0	0	0	0	0	0	0	1 (5.6)	0	0	0
	Grade 3-Severe	0	0	0	0	0	0	0	0	0	0	0	0
Blurred Vision	Reported*							0	0	0	0	1 (20.0)	0
	Grade 1-Mild							0	1 (5.6)	0	0	0	0
	Grade 2-Moderate							0	0	0	0	0	0
	Grade 3-Severe							0	0	0	0	0	0
Chest Pains	Grade 1-Mild							0	0	0	0	0	0
	Grade 2-Moderate							0	0	0	0	0	0
	Grade 3-Severe							0	0	0	0	0	0
Chills	Reported*	0	0	0	0	0	0	0	0	1 (5.6)	0	0	0
	Grade 1-Mild	0	0	0	0	0	0	0	0	0	0	0	0
	Grade 2-Moderate	0	0	0	0	0	0	0	0	0	0	0	0
	Grade 3-Severe	0	0	0	0	0	0	0	0	0	0	0	0

		PfSPZ Vaccine						PfSPZ-CVac					
		$2.7x10^6$			Placebo			$1.0x10^5$			Placebo		
**Solicited Event**	**Grade**	**Dose 1 N=20**	**Dose 2 N=18**	**Dose 3 N=18**	**Dose 1 N=6**	**Dose 2 N=6**	**Dose 3 N=6**	**Dose 1 N=19**	**Dose 2 N=18**	**Dose 3 N=18**	**Dose 1 N=5**	**Dose 2 N=5**	**Dose 3 N=4**
Confusion	Grade 1-Mild							0	0	0	0	0	0
	Grade 2-Moderate							0	0	0	0	0	0
	Grade 3-Severe							0	0	0	0	0	0
Cough	Grade 1-Mild							0	1 (5.6)	0	0	0	0
	Grade 2-Moderate							0	0	0	0	0	0
	Grade 3-Severe							0	0	0	0	0	0
Diarrhea	Grade 1-Mild							0	1 (5.6)	0	0	0	0
	Grade 2-Moderate							0	0	1 (5.6)	0	0	0
	Grade 3-Severe							0	0	0	0	0	0
Dizziness	Reported*							1 (5.3)	0	0	2 (40.0)	0	0
	Grade 1-Mild							0	1 (5.6)	0	1 (20.0)	0	0
	Grade 2-Moderate							0	0	0	0	0	0
	Grade 3-Severe							0	0	0	0	0	0
Fatigue	Reported*	1 (5.0)	0	1 (5.6)	0	0	0	1 (5.3)	0	0	0	0	0
	Grade 1-Mild	2 (10.0)	0	1 (5.6)	0	0	0	0	0	1 (5.6)	1 (20.0)	0	0
	Grade 2-Moderate	0	0	0	0	0	0	0	0	0	0	0	0
	Grade 3-Severe	0	0	0	0	0	0	0	0	0	0	0	0
Fever	Grade 1-Mild	0	0	0	0	0	0	0	1 (5.6)	0	0	0	0
	Grade 2-Moderate	0	0	0	0	0	0	0	0	0	0	0	0
	Grade 3-Severe	0	0	0	0	0	0	0	0	0	0	0	0

		PfSPZ Vaccine						PfSPZ-CVac					
		$2.7x10^6$			Placebo			$1.0x10^5$			Placebo		
**Solicited Event**	**Grade**	**Dose 1 N=20**	**Dose 2 N=18**	**Dose 3 N=18**	**Dose 1 N=6**	**Dose 2 N=6**	**Dose 3 N=6**	**Dose 1 N=19**	**Dose 2 N=18**	**Dose 3 N=18**	**Dose 1 N=5**	**Dose 2 N=5**	**Dose 3 N=4**
Headache	Reported*	1 (5.0)	0	1 (5.6)	0	0	0	1 (5.3)	0	0	0	0	0
	Grade 1-Mild	0	0	0	0	0	0	2 (10.5)	1 (5.6)	0	1 (20.0)	0	0
	Grade 2-Moderate	1 (5.0)	0	0	0	0	0	0	2 (11.1)	0	0	0	0
	Grade 3-Severe	0	0	0	0	0	0	0	0	0	0	0	0
Insomnia	Grade 1-Mild							0	1 (5.6)	0	0	0	0
	Grade 2-Moderate							0	0	0	0	0	0
	Grade 3-Severe							0	0	0	0	0	0
Malaise	Grade 1-Mild	0	0	0	0	0	0	0	0	0	0	0	0
	Grade 2-Moderate	0	0	0	0	0	0	0	0	0	0	0	0
	Grade 3-Severe	0	0	0	0	0	0	0	0	0	0	0	0
Myalgia	Reported*	1 (5.0)	0	0	0	0	0	0	0	0	0	0	0
	Grade 1-Mild	2 (10.0)	0	0	0	0	0	0	0	0	1 (20.0)	1 (20.0)	0
	Grade 2-Moderate	0	0	0	0	0	0	0	0	0	0	0	0
	Grade 3-Severe	0	0	0	0	0	0	0	0	0	0	0	0
Nausea	Reported*							0	0	0	0	1 (20.0)	0
	Grade 1-Mild							1 (5.3)	0	0	0	0	0
	Grade 2-Moderate							0	0	0	0	0	0
	Grade 3-Severe							0	0	0	0	0	0
Palpitations	Grade 1-Mild							0	0	0	0	0	0
	Grade 2-Moderate							0	0	0	0	0	0
	Grade 3-Severe							0	0	0	0	0	0

		PfSPZ Vaccine						PfSPZ-CVac					
		2.7x10[6]			Placebo			1.0x10[5]			Placebo		
**Solicited Event**	**Grade**	**Dose 1 N=20**	**Dose 2 N=18**	**Dose 3 N=18**	**Dose 1 N=6**	**Dose 2 N=6**	**Dose 3 N=6**	**Dose 1 N=19**	**Dose 2 N=18**	**Dose 3 N=18**	**Dose 1 N=5**	**Dose 2 N=5**	**Dose 3 N=4**
Photosensitivity	Grade 1-Mild							0	0	0	0	0	0
	Grade 2-Moderate							0	0	0	0	0	0
	Grade 3-Severe							0	0	0	0	0	0
Pruritus	Reported*							1 (5.3)	0	0	0	0	0
	Grade 1-Mild							1 (5.3)	0	0	0	0	0
	Grade 2-Moderate							0	0	0	0	0	0
	Grade 3-Severe							0	0	0	0	0	0
Rash, urticaria, pruritus, edema	Reported*	0	0	0	0	0	0	1 (5.3)	0	0	0	0	0
	Grade 1-Mild	0	0	0	0	0	0	1 (5.3)	0	0	0	0	0
	Grade 2-Moderate	0	0	0	0	0	0	0	0	0	0	0	0
	Grade 3-Severe	0	0	0	0	0	0	0	0	0	0	0	0
Rigors	Grade 1-Mild							0	0	0	0	0	0
	Grade 2-Moderate							0	0	0	0	0	0
	Grade 3-Severe							0	0	0	0	0	0
Shortness of Breath	Grade 1-Mild							0	0	0	0	0	0
	Grade 2-Moderate							0	0	0	0	0	0
	Grade 3-Severe							0	0	0	0	0	0
Subjective Fever	Reported*	0	0	1 (5.6)	0	0	0	0	0	0	0	0	0
	Grade 1-Mild	1 (5.0)	1 (5.6)	0	0	0	0	1 (5.3)	1 (5.6)	0	0	0	0
	Grade 2-Moderate	0	0	0	0	0	0	0	0	0	0	0	0
	Grade 3-Severe	0	0	0	0	0	0	0	0	0	0	0	0

		PfSPZ Vaccine						PfSPZ-CVac					
		$2.7x10^6$			Placebo			$1.0x10^5$			Placebo		
**Solicited Event**	**Grade**	**Dose 1 N=20**	**Dose 2 N=18**	**Dose 3 N=18**	**Dose 1 N=6**	**Dose 2 N=6**	**Dose 3 N=6**	**Dose 1 N=19**	**Dose 2 N=18**	**Dose 3 N=18**	**Dose 1 N=5**	**Dose 2 N=5**	**Dose 3 N=4**
Sweats	Grade 1- Mild							0	0	0	0	0	0
	Grade 2- Moderate							0	0	0	0	0	0
	Grade 3- Severe							0	0	0	0	0	0
Tinnitus	Grade 1- Mild							0	0	0	1 (20.0)	0	0
	Grade 2- Moderate							0	0	0	0	0	0
	Grade 3- Severe							0	0	0	0	0	0
Vomiting	Grade 1- Mild							0	0	0	0	0	0
	Grade 2- Moderate							0	0	0	0	0	0
	Grade 3- Severe							0	0	0	0	0	0

Denominators are based on the number of subjects with systemic solicited event records submitted for each vaccine dose at the time of data cutoff
*Symptom was reported but grading was not done.

Table S7: Abnormal Laboratory Values[a]. Number (and %) of subjects in each group experiencing the listed lab abnormality at least one during the study period.

	Group 1A		Group 1B	
**Lab parameter**	**2.7x10^6 (N=20)**	**Placebo (N=6)**	**1.0x10^5 (N=19)**	**Placebo (N=5)**
Red Blood Cells	0 (0.0)	0 (0.0)	0 (0.0)	0 (0.0)
Decreased Hemoglobin	2 (10.0)	2 (33.3)	3 (15.8)	0 (0.0)
Decreased Platelets	2 (10.0)	1 (16.7)	2 (10.5)	1 (20.0)
Increased WBC Count	0 (0.0)	0 (0.0)	1 (5.3)	2 (40.0)
Decreased WBC Count	7 (35.0)	3 (50.0)	9 (47.4)	0 (0.0)
Decreased Neutrophils	15 (75.0)	4 (66.7)	18 (94.7)[†]	2 (40.0)
Decreased Lymphocytes	3 (15.0)	2 (33.3)	5 (26.3)	0 (0.0)
Increased Eosinophils	7 (35.0)	3 (50.0)	9 (47.4)	3 (60.0)
Elevated ALT	2 (10.0)	3 (50.0)	4 (21.1)	2 (40.0)
Elevated AST	3 (15.0)	1 (16.7)	5 (26.3)	2 (40.0)
Elevated Total Bilirubin	0 (0.0)	0 (0.0)	1 (5.3)*	0 (0.0)
Elevated Creatinine	4 (20.0)	1 (16.7)	3 (15.8)	0 (0.0)
Hypoglycemia	0 (0.0)	0 (0.0)	0 (0.0)	0 (0.0)

*Includes at least one Grade 3 result.
[†]p=0.0089, Barnard's test, 2-tailed. No other comparison between vaccine and corresponding control was staitistically significant.

Table S8: Asymptomatic parasitemia detected during the study prior to CHMI.

Study number	Group or prospective group	Vaccine	Time point(s)	Species
408	PfSPZ Vaccine	$2.7x10^{6}$ PfSPZ	Screening	*P. malariae*
			V2	*P. falciparum*
			CHMI-7	*P. falciparum*
			CHMI	*P. falciparum, P. malariae*
415	PfSPZ Vaccine	placebo	V3, V3+28, V3+56	*P. malariae*
416	PfSPZ Vaccine	$2.7x10^{6}$ PfSPZ	V3	*P. ovale*
			V3+196	*P. falciparum*
431	PfSPZ Vaccine	placebo	Scr3	*P. falciparum, P. malariae*
			V2	*P. malariae*
			V3, V3+28, V3+56	*P. falciparum*
404	PfSPZ-CVac	$1.0x10^{5}$ PfSPZ	CHMI-7	*P. falciparum*
512	PfSPZ-CVac	$1.0x10^{5}$ PfSPZ	CHMI-7, CHMI-7 (2)	*P. falciparum**
515	PfSPZ-CVac	placebo	CHMI	*P. falciparum**
519	PfSPZ-CVac	$1.0x10^{5}$ PfSPZ	Sc3	*P. falciparum*
525	PfSPZ-CVac	$1.0x10^{5}$ PfSPZ	V3, V3+14, CHMI-7	*P. falciparum**
528	PfSPZ-CVac	$1.0x10^{5}$ PfSPZ	CHMI-7	*P. falciparum, P. ovale*
530	PfSPZ-CVac	$1.0x10^{5}$ PfSPZ	CHMI-7	*P. falciparum*

* - genotyping confirmed as wild type or not the PfSPZ Challenge strain (NF54).
For the remaining Pf isolates in the PfSPZ-CVac arm, genotyping was either not performed (2) or inconclusive (2). In the PfSPZ Vaccine arm, all Pf infections were assumed to be naturally acquired field strains.

Table S9: Genotype data:

sample metadata			malaria qPCR data		msp1/msp2 genoptyping					drug resistance marker			microsatellite genotyping						Conclusion
**Sid**	**grp**	**visit**	**pf/uL**	**Non-Pf**	**msp1_k**	**msp1_m**	**msp1_r**	**msp2_fc**	**msp2_ic**	**k13**	**dhfr**	**dhps**	**Poly-A**	**PFPK2**	**TA-81**	**ARA-2**	**TA-87**	**TA-40**	
culture derived NF54			-	-	250	-	-	-	500	PFNF 54	PFNF54	PFNF54	153	172	123	67	100	223	PfNF54
512	G1B	PD	0.35	-	-	200	-	-	-	-	-	-	-	-	-	-	-	-	field strain
525	G1B	PD	10.1	-	200	-	-	-	500; 600	PFNF 54	N51I; C59R; S108N	PFNF54	-	-	-	-	-	-	multiple strain infection, PfNF54 unlikley
525	G1B	CH-7	22.1	-	200	-	-	-	500; 600	PFNF 54	N51I; C59R; S108N	PFNF54	-	-	-	-	-	-	field strain
528	G1B	CH-7	92.2	Po	250; 400	200	-	350	500	PFNF 54	N51I; C59R; S108N	PFNF54	-	-	-	-	-	-	multiple strain infection, PfNF54 can NOT be excluded
529	G1B	CH-7	0.9	-	-	-	-	-	-	-	-	-	-	-	-	-	-	-	inconclusive
530	G1B	CH-7	0.1	-	-	-	-	-	-	-	-	-	-	-	-	-	-	-	inconclusive
510	G1B	CH-7	0.25	-	-	-	-	-	-	-	-	-	-	-	124	-	-	-	inconclusive
515	G1B	PD	84.9	-	-	200	-	-	700	PFNF 54	N51I; C59R; S108N	S436A; G437A	-	164	-	70	109	-	field strain
316	G1A	CH+18 T0	63.2	-	250	-	-	-	500	PFNF 54	PFNF54	PFNF54	-	-	122	-	-	-	PfNF54
314	G1A	CH+18 T0	25	-	250	-	-	-	500	PFNF 54	PFNF54	-	-	-	122	-	-	-	PfNF54
303	G1A	CH+13 T0	59.3	-	250	-	-	-	500	PFNF 54	PFNF54	PFNF54	-	-	-	67	100	-	PfNF54
309	G1A	CH+18 T0	53.5	-	250	-	-	-	500	PFNF 54	PFNF54	PFNF54	-	-	122	-	-	-	PfNF54

Table S10. Solicited AE, unsolicited AE and symptoms and signs of malaria. Solicited AE were collected for 5 days after CHMI. Specific symptoms and signs of malaria were solicited at each visit starting at day 7 through to day 29 and were attributed to malaria if they corresponded to Pf parasitemia as described. Unsolicited AE not corresponding to parasitemia and presumed unrelated to malaria were collected from days 1 to 29.

	All (n=36)	TBS+/qPCR+ (n=15)	TBS-/qPCR+ (n=6)	TBS-/qPCR- (n=15)
Number subjects (%) with solicited AEs, CHMI days 1-6	1 (2.8%)			
Number of subjects with symptoms or signs of malaria*	9 (25.0%)	8 (53.3%)	1 (16.7%)	0 (0.0%)
Number subjects with unsolicited AEs, CHMI days 1-29[#]	7 (19.4%)	2 (13.3%)	2 (33.3%)	3 (20.0%)

*Symptoms or signs of malaria were identified using a predefined list of symptoms or signs occurring from 3 days prior to 7 days after the detection of parasitemia by TBS. For the one qPCR+/TBS- subject with symptoms the identified symptoms occurred beginning 5 days after the first positive sample was positive by qPCR.

[#]Unsolicited AE included toothace (3), arthralgias, conjunctivitis, left foot swelling, nipple pain, trauma to the right great toe and upper lip swelling. None were considered related to injection of PfSPZ Challenge.
